# Serglycin secreted by late-stage nucleus pulposus cells is a biomarker of intervertebral disc degeneration

**DOI:** 10.1038/s41467-023-44313-9

**Published:** 2024-01-02

**Authors:** Fan Chen, Linchuan Lei, Shunlun Chen, Zhuoyang Zhao, Yuming Huang, Guowei Jiang, Xingyu Guo, Zemin Li, Zhaomin Zheng, Jianru Wang

**Affiliations:** 1 Department of Spine Surgery, The First Affiliated Hospital of Sun Yat-sen University, Guangzhou, 510080 P.R. China; 2 Guangdong Province Key Laboratory of Orthopaedics and Traumatology, Guangzhou, 510080 P.R. China; 3 Laboratory of General Surgery, The First Affiliated Hospital, Sun Yat-sen University, Guangzhou, 510080 China

**Keywords:** Diseases, Mechanisms of disease, Chronic inflammation, Predictive markers

## Abstract

Intervertebral disc degeneration is a natural process during aging and a leading cause of lower back pain. Here, we generate a comprehensive atlas of nucleus pulposus cells using single-cell RNA-seq analysis of human nucleus pulposus tissues (three males and four females, age 41.14 ± 18.01 years). We identify fibrotic late-stage nucleus pulposus cells characterized by upregulation of serglycin expression which facilitate the local inflammatory response by promoting the infiltration of inflammatory cytokines and macrophages. Finally, we discover that daphnetin, a potential serglycin ligand, substantially mitigates the local inflammatory response by downregulating serglycin expression in an in vivo mouse model, thus alleviating intervertebral disc degeneration. Taken together, we identify late-stage nucleus pulposus cells and confirm the potential mechanism by which serglycin regulates intervertebral disc degeneration. Our findings indicate that serglycin is a latent biomarker of intervertebral disc degeneration and may contribute to development of diagnostic and therapeutic strategies.

## Abstract (web summary)

Aging-related intervertebral disc degeneration (IVDD) is a leading cause of lower back pain. Here, the authors perform scRNA-seq analysis of intervertebral disc cells from patients, and identify cell populations and mechanisms associated with IVDD.

## Introduction

Intervertebral disc degeneration (IVDD), a common process during natural aging, results in severe spinal symptoms, notably lower back pain (LBP), and has emerged as a leading cause of disability worldwide^1,2^. Previous studies have shown that the degeneration and progression of the local inflammatory response in intervertebral disc (IVD) is important in IVDD^3,4^. However, the mechanisms underlying the elevated local inflammatory response and IVDD remain unclear.

The IVD consists of a gel-like nucleus pulposus (NP) that is circumferentially surrounded by a fibrocartilaginous annulus fibrosus (AF) and vertically surrounded by cartilaginous endplates (CEPs), which help sustain the natural flexibility and normal biomechanical functioning of the spine^5^. Researchers have confirmed that the abnormal function of any component of IVD results in IVDD, and NP tissue degeneration is the main cause of IVDD^6,7^. Although NP cells exhibit heterogeneity, nucleus pulposus cells (NPCs) is the primary cell type. The divergence of NPCs plays an important role in the progression of IVDD. However, the detailed subpopulations and cell-type-specific biomarkers of heterogeneous NPCs remain to be elucidated.

In recent years, single-cell RNA sequencing (scRNA-seq) has become an increasingly popular and powerful method to evaluate single-cell gene expression and to identify heterogeneous subpopulations within a tissue, providing an option to map the types, subsets, and states of cells in healthy and unhealthy conditions in an unprecedented manner, along with providing insights into physiological and pathological processes. ScRNA-seq was initially used in IVD research. Few related studies have identified novel phenotypic biomarkers of NP cells and provided new insights into IVD cell heterogeneity^8–14^. Hence, higher-resolution investigations are needed to determine the phenotypic markers of the rare subpopulations of NPCs and to explore their potential functions in the process of IVDD, as well as the underlying mechanisms, to elucidate the physiology and pathology of IVDD and develop new therapies.

Serglycin (SRGN) is an intracellular, low-molecular-weight glycoprotein that is widely distributed in diverse cell types and integrated into the extracellular matrix (ECM)^15^. SRGN plays important roles in many physiological processes by storing and releasing cytokines, chemokines, and proteases^16^. Previous studies have shown that SRGN plays a role in inflammation and the progression of multiple cancers^17^. Michele et al. reported that SRGN is associated with the inflammatory response of human chondrocytes exposed to interleukin (IL)−1β and the CD44 receptor^18^. Wang et al. found that SRGN expression had a significant positive correlation with macrophage infiltration^19^. Many studies have suggested that exogenous treatment with lipopolysaccharide (LPS), tumor necrosis factor (TNF)-α, and IL-1β can induce SRGN overexpression in different cell types^20–23^. Giving that the local inflammatory response is one of the main causes of IVDD^24,25^, it remains unclear whether SRGN regulates the local inflammatory response in IVDs and its mechanism in IVDD.

Daphnetin (DAP) is a natural coumarin derivative extracted from plants of the genus *Daphne*. This molecule has been reported to exhibit anti-apoptotic, anticancer, anti-macrophage infiltration and, in particular, anti-inflammatory properties^26–28^. DAP is used to treat several diseases, including lung injury, rheumatoid arthritis, inflammatory pain, severe acute pancreatitis, and osteoarthritis^28–30^. DAP has been synthesized and incorporated into drugs for treating thromboangiitis obliterans and other occlusive vascular diseases^31^. Nevertheless, the specific impact of DAP on IVDD remains unclear.

**Fig. 1:**
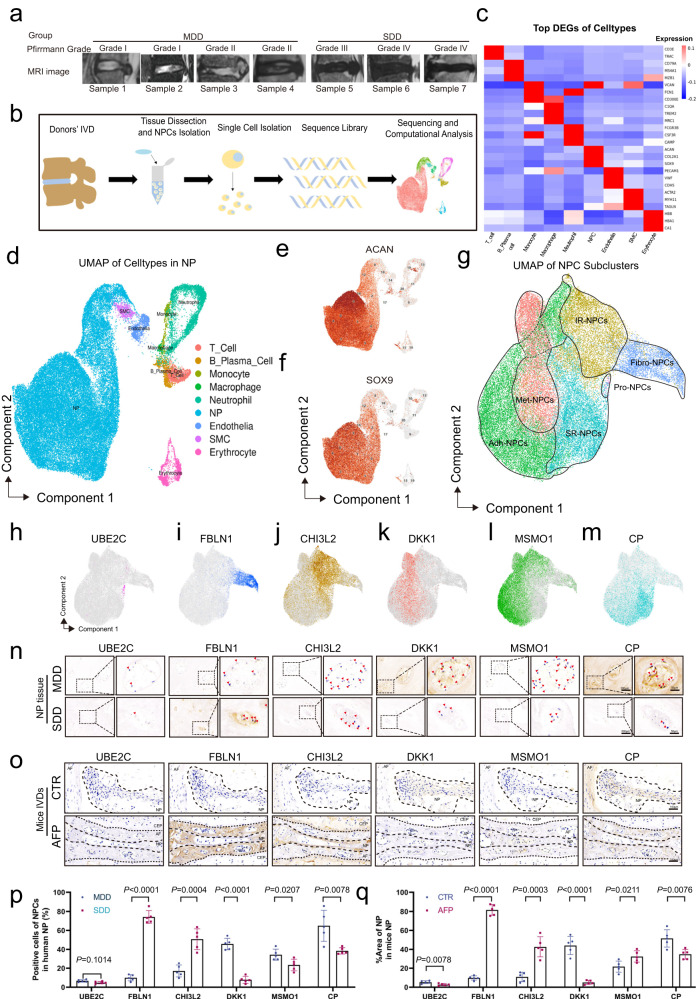
scRNA-seq atlas of human NP tissues identifies six cell clusters. **a** T2 MRI image of 7 samples of a single-cell sequence. (mild degenerative disc [MDD]: *n* = 4, severe degenerative disc [SDD]: *n* = 3). **b** Schematic workflow of the experimental design. Cells isolated from the NP of human IVDs were subjected to scRNA-seq. **c** Heatmap of marker genes of the 9 cell types. **d** UMAP map of the following 9 cell types: T cells, B cells, monocytes, macrophages, neutrophils, NPCs, endothelial cells, SMCs, and erythrocytes. **e**, **f** UMAP image of the NPC marker genes: *ACAN* and *SOX9*. **g** A single-cell atlas revealed the cell distributions and cell type distributions of NPCs. **h**–**m** The differentially expressed genes in each of the 6 NPC subclusters. **n**–**q** Representative IHC image with marker gene (*UBE2C*, *FBLN1*, *CHI3L2*, *DKK1*, *MSMO1* and *CP*) expression of the 6 NPC clusters in MDD and SDD human tissue (**n**, **p**, scale bar: 200 µm left and 50 µm right panels) and in CTR and AFP group model mouse IVDs (**o**, **q**, scale bar: 200 µm up and 50 µm down panels). MDD mild degenerative discs, SDD severe degenerative discs, DEGs differentially expressed genes, UMAP uniform manifold approximation and projection, SMCs smooth muscle cells, red arrows: NPCs; CTR control groups, AFP annulus fibrosus puncture group, AF annulus fibrosus, CEP cartilaginous end plate, NP nucleus pulposus, IVD intervertebral disc. *n* = 5 each group. Data are presented as mean ± SD. Statistical significance was determined by two-tailed unpaired *t* test. Source data are provided as a Source Data file.

**Fig. 2:**
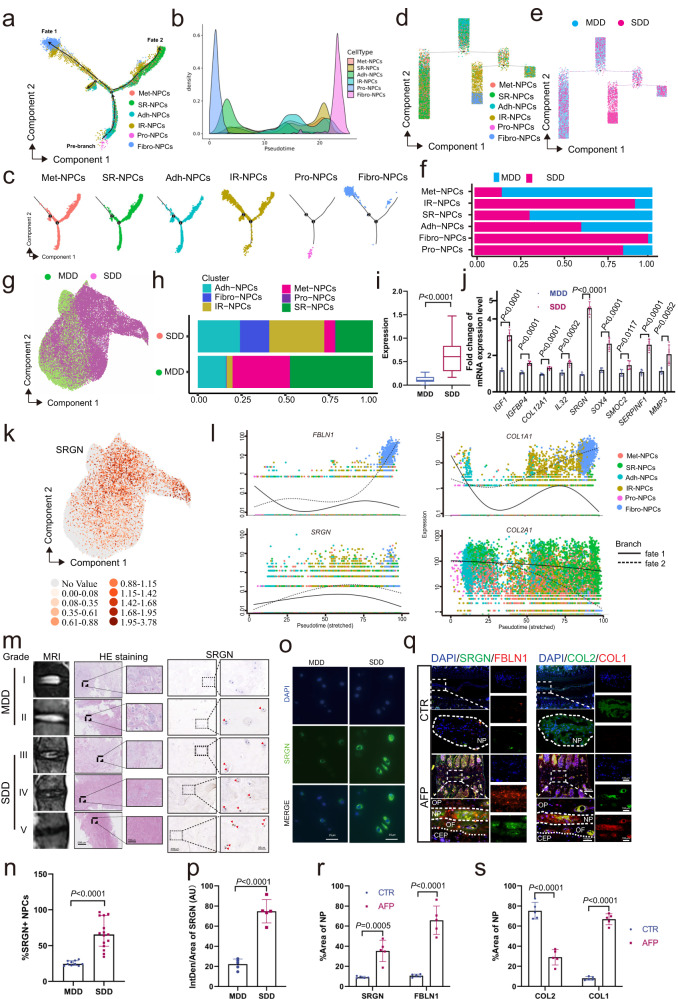
Identification of late-stage NPCs with upregulated SRGN expression. **a**–**c** Monocle pseudotime trajectory showing the progression of Met-, SR-, Adh- IR-, Pro- and Fibro-NPCs. **d** Tree plot of the monocle pseudotime trajectory showing the progression of 6 subclusters of NPCs. **e** Tree plot of the monocle pseudotime trajectory showing the progression of MDD and SDD NPCs. **f**–**h** A single-cell atlas revealed the cell distributions of NPCs in the MDD and SDD groups (**g**), the subcluster composition of NPCs (**f**), and the proportions of each subcluster in MDD and SDD NPCs (**h**). **i** *SRGN* expression in MDD and SDD tissues by scRNA-seq analysis. **j** The screening RT‒qPCR results showed the expression of candidate marker genes of degenerative NPCs. **k** UMAP plot of *SRGN* expression. **l** Pseudotime kinetics of *SRGN*, *FBLN1*, *COL1A1* and *COL2A1* in each subcluster of NPCs. **m**, **n** MRI images and Pfirrmann grades of human discs and H&E and IHC staining of SRNG expression in different degenerative human NP specimens (red arrows: SRGN+ NPCs; original magnification ×100, ×400, scale bar = 200 µm, 50 µm). **o**, **p** IF staining of SRGN (green) in normal NP samples compared to degenerated NP samples (original magnification ×400, scale bar = 50 µm). **q**–**s** IF staining of the late-stage NPC markers FBLN1 (red) and SRGN (green) and the degenerative indicators COL1A (red) and COL2A1 (green) in tissue samples from mouse IVDs (original magnification ×100, ×400, scale bar = 400 μm, 100 µm). MDD mild degenerative discs, SDD severe degenerative discs, CTR control group, AFP annulus fibrosus puncture group, AF annulus fibrosus, CEP cartilaginous end plate, NP nucleus pulposus. *n* = 5 each group. Data in 2i are presented as the median±interquartile range as appropriate, and the *p* value was determined by the unpaired Wilcox rank sum test. Data in (**j**) and (**m**–**s**) are presented as the mean ± standard deviation, and *p* values were determined by a two-tailed unpaired *t* test. Source data are provided as a Source Data file.

Building upon the study above, we used scRNA-seq to generate a comprehensive atlas of NPCs. We annotated 20 cell populations through differential gene expression analysis (DGEA) of each cluster (Supplementary Fig. 2a). Then, the graph cluster and K-mean were utilized for cell clustering and the Wilcoxon rank sum test was used for marker gene analysis. We annotated clusters into 9 populations (Supplementary Fig. 2b, Fig. 2d). The heterogeneity of NPCs was classified by gene expression (*SOX9* and *ACAN*) and the functional analysis (Fig. 2e, f)^32^, NPCs were further classified into six subpopulations based on their marker genes *UBE2C*, *FBLN1*, *CH3L2*, *DKK1*, *MSMO1*, and *CP* (Figs. 1, 2g–m). Transcriptional entropy scoring and pseudotime analysis were performed to assess the fate characteristics of each subpopulation and resolve differentiation lineages. Fibro-NPCs were positioned at the terminal point of the pseudotime trajectory, which was defined as the late-stage NPCs, playing essential role in IVDD. Interestingly, we observed an upregulation of *SRGN* in degenerative NPCs, especially in late-stage NPCs. Subsequently, we found that SRGN plays an important role in IVDD by regulating the IVD local inflammatory response. Finally, we investigated whether DAP could downregulate the expression of SRGN in late-stage NPCs, thereby alleviating IVDD. Thus, the objective of this study was to identify and characterize NPC subpopulations that expedite the advancement of IVDD. In addition, we aimed to determine the gene expression, functional roles, and mechanisms of late-stage NPCs in the IVDD process. We found that the administration of DAP could slow the IVDD by downregulating SRGN expression in vivo and in vitro.

## Results

### scRNA-seq atlas of human NP tissues identifies six cell clusters

To investigate the subpopulations of NPCs and the gene features of human NPCs during IVDD, we collected human NP tissue samples from patients undergoing discectomy at the First Affiliated Hospital of Sun Yat-Sen University. These samples were divided into mild (grades I-II) and severe (grades III-V) degenerative NP tissues according to the Pfirrmann grading system (Fig. 1a). ScRNA-seq analysis was performed based on the 10X Genomics platform (Fig. 1b, Supplementary Table 1) (mild degenerative disc [MDD]: *n* = 4, two males and two females; severe degenerative disc [SDD]: *n* = 3, one male and two females; age 41.14 ± 18.01 years). Representative T2 magnetic resonance imaging (MRI) images are shown in Fig. 1a. Cell filtration information is shown in Supplementary Fig. 1.

Utilizing unsupervised graph-based clustering of the integrated dataset comprised of 55,264 cells from the seven samples, we identified 20 distinct clusters based on nearest neighbor approximations^33^ (Supplementary Fig. 2a). Then, Graphcluster and K-mean were employed for cell clustering, and the Wilcox rank sum test was used for marker gene analysis (Supplementary Fig. 2 b). Using uniform manifold approximation and projection (UMAP) plots, hierarchical clustering, and gene expression signatures of published canonical markers (Fig. 1c), we annotated clusters into the following population partitions: nucleus pulposus cells (NPCs, *ACAN*+ and *SOX9*+), T cells, B cells, macrophages, neutrophils, endothelial cells, smooth muscle cells (SMCs), and erythrocytes (Fig. 1d). The heterogeneity of NPCs was further classified by gene expression (*SOX9* and *ACAN*) and functional analysis (Fig. 1e, f, Supplementary Fig. 3), and the representative gene markers are shown in Fig. 1h–m with the feature plot map. Subsequently, six subtypes of NPCs were further identified based on their marker genes *UBE2C*, *FBLN1*, *CH3L2*, *DKK1*, *MSMO1*, and *CP* (unsupervised clustering algorithms) (Fig. 1g–m). These subtypes were designated as follows (Fig. 1g):

Pro-NPCs: progenitor NPCs, marked with *ACAN*, *SOX9*, *UBE2C,* and *TOP2A*; Fibro- NPCs: fibrotic NPCs, marked with *ACAN*, *SOX9*, *FBLN1*^34,35^; IR-NPCs: integral regulatory NPCs, marked with *ACAN*, *SOX9*, *CH3L2*^36^; Met-NPCs: metabolic NPCs, marked with *ACAN*, *SOX9*, *DKK1*^37^; Adh-NPCs: adhesive NPCs, marked with *ACAN*, *SOX9,* and *MSMO1*^36^; and SR-NPCs: stress-responsive NPCs, marked with *ACAN*, *SOX9,* and *CP*.

Furthermore, immunohistochemical (IHC) staining and corresponding quantitative analysis of human and mouse NP tissues illustrated the distribution of each cluster of NPCs and the homologous comparison between humans and mice (Fig. 1n–q). These results described the characteristics and six subclusters of NPCs.

### Identification of late-stage NPCs with upregulated SRGN expression

To investigate the functional role of NPC subpopulations during IVDD, pseudotime analysis (Monocle 2) was conducted on the 6 clusters of NPCs (*ACAN*+ and *SOX9*+ cells). The results suggested that Pro-NPCs were at the initial stage of the pseudotime trajectory, while Fibro-NPCs were at the end and were mainly distributed in the late stage. While IR-NPCs present widespread distribution across all stages; Met-, SR-, and Adh-NPCs exhibited a similar trend in the trajectory analysis, suggesting a potential shared fate among these subpopulations. (Fig. 2a–c, Supplementary Fig. 4a). Simultaneously, the proportions of each cell cluster in MDD and SDD NP tissues were analyzed, and the Fibro-NPC cluster was also mainly distributed in the severe degenerative group and closely related to IVDD (Fig. 2d–h). The Gene Ontology (GO) analysis of each cluster revealed that Fibro-NPCs were involved in the collagen fibril and ECM organization of NPCs (Supplementary Fig. 3). As described in previous studies, *FBNL1* (a marker gene of Fibro-NPCs) also participated in collagen fibrils, ECM organization and cell fates (such as cell apoptosis and senescence)^35,38^. Thus, according to the scRNA-seq, pseudotime analysis and functional analysis results, the Fibro-NPCs were characterized as late-stage NPCs, one kind of cell phenotype of the NPCs.

To explore gene expression changes and to identify markers for late-stage NPCs, scRNA-seq analysis was applied, the results showed enhanced expression of *SRGN* in the SDD group, especially in late-stage NPCs (Fig. 2i and Supplementary Fig. 4b). In addition, RNA-Seq analysis (MDD: *n* = 3, two males and one female; SDD: *n* = 3, two males and one female; age 56.83 ± 7.33 years) showed that *SRGN* was upregulated in the SDD group and was positively correlated with IVDD-related markers (Supplementary Fig. 4c–e). Furthermore, a combined analysis of the top 100 upregulated genes in RNA-Seq and late-stage NPCs highlighted SRGN as one of the 9 common genes (*IGF1*, *IGFBP4*, *COL12A1*, *IL32*, *SRGN*, *SOX4*, *SMOC2*, *SERPINF1*, *MMP3*) (Supplementary Fig. 4f). Subsequently, the mRNA expression levels of the nine selected genes were measured in human NP tissue, and *SRGN* was markedly increased (Fig. 2j). Furthermore, the UMAP distribution of *SRGN* expression in NPCs is shown in the scatter plot (Fig. 2k). Monocle trajectory analysis and pseudotime dynamic expression analysis showed significantly increased expression of *SRGN* in late-stage NPCs; *SRGN*, *FBLN1* and *COL1A1* had similar dynamic trends, and *COL2A1* had the opposite trend (Fig. 2l).

Subsequently, human IVDs with various degrees of degeneration were identified by MRI, as shown in Fig. 2m (grades I, II, III, IV, and V). Hematoxylin and eosin (H&E) staining showed that the number of NPCs (red arrows) was decreased in the SDD group. IHC (Fig. 2m, n) and immunofluorescence (IF) staining (Fig. 2o, p) revealed SRGN overexpression in the SDD group compared to that in the MDD group. Western blotting analysis suggested that SRGN expression was significantly upregulated in the SDD group compared to the MDD group (Supplementary Fig. 4g). Furthermore, IF staining revealed simultaneous upregulated expression of SRGN, FBLN1 (a marker of Fibro-NPCs) and COL1A1 in the IVDD mouse model (Fig. 2q–s). These findings collectively confirmed the identification of late-stage NPCs and the upregulation of SRGN specifically in late-stage NPCs during the progression of IVDD.

### Inhibition of SRGN could alleviate the progression of IVDD

**Fig. 3:**
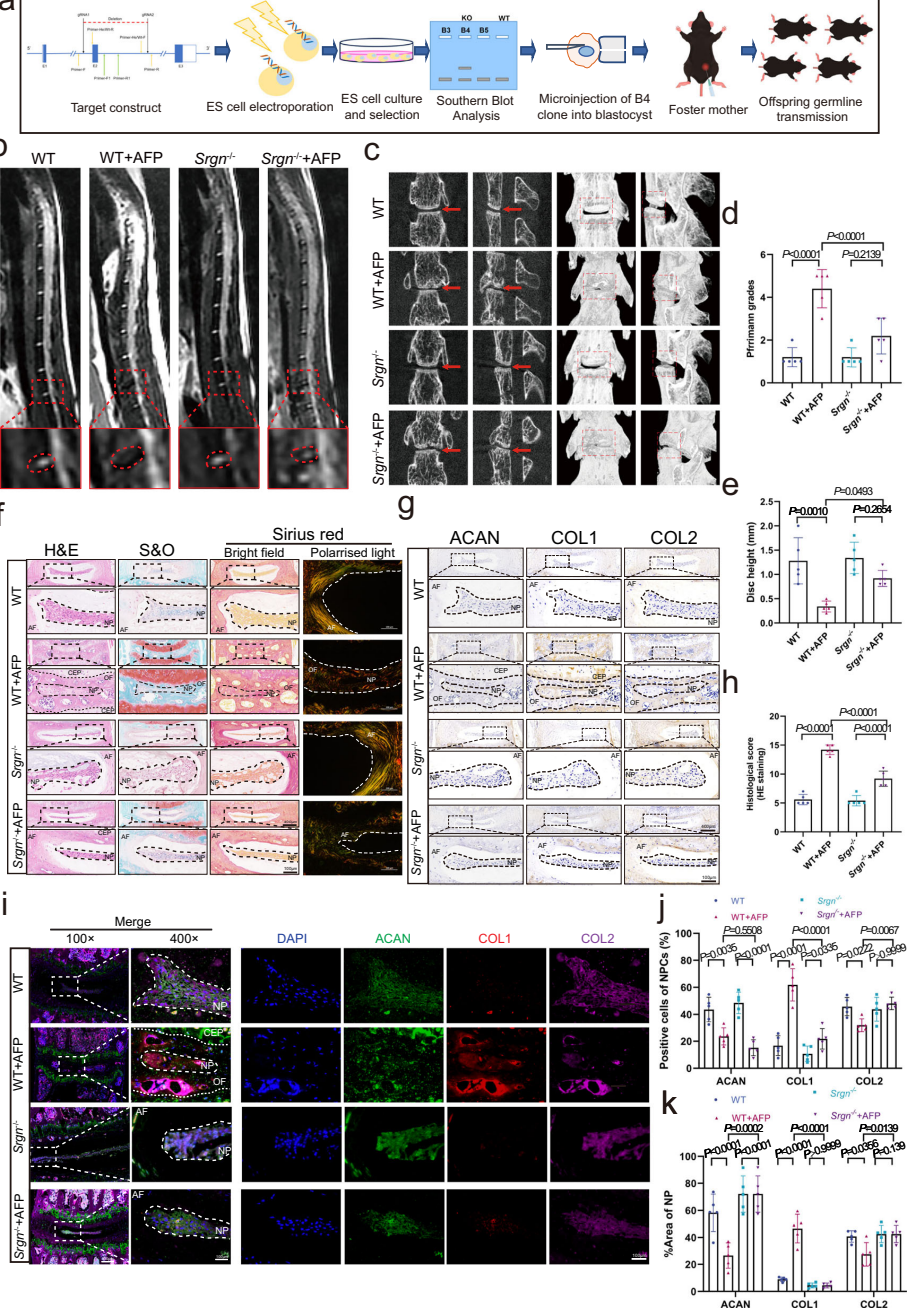
Inhibition of SRGN alleviates the progression of IVDD. **a** Flow diagram for establishing *Srgn*^−/−^ mice. **b**, **d** MRI images and Pfirrmann grade analysis of *Srgn*^−/−^ mice and IVDD models at 8 weeks after operation. **c**, **e** Micro-CT images and disc height analysis of *Srgn*^−/−^ mice and IVDD models 8 weeks after operation. **f**, **h** H&E staining, Safranin-O staining, and bright field Sirius red staining with polarized light images of IVD tissue from *Srgn*^−/−^ mice and IVDD models 8 weeks after operation (original magnification ×100, ×400, scale bar = 400 µm, 100 µm). **g**, **j** IHC staining for COL1, COL2, and ACAN in WT, *Srgn*^−/−^, WT plus AF, and *Srgn*^−/−^ plus AF mice 8 weeks after operation (original magnification ×100, ×400, scale bar = 400 µm, 100 µm). **i**, **k** IF staining of COL1 (red), COL2 (purple), and ACAN (green) in WT plus AF and *Srgn*^−/−^ plus AF mice 8 weeks after operation (original magnification ×100, ×400, scale bar = 400 µm, 100 µm). CTR control group, AFP annulus fibrosus puncture group, AF annulus fibrosus, CEP cartilaginous end plate, NP nucleus pulposus. *n* = 5 each group. Data are represented as mean ± standard deviation. *P* values were determined by one-way ANOVA with post hoc Bonferroni correction or Kruskal-Wallis *H* test with a Dunn’s correction as appropriate. Source data are provided as a Source Data file.

To confirm the critical role of SRGN in IVDD, *SRGN* knockout mice were established (Supplementary Fig. 5), and subjected to AF puncture, MRI, and microcomputed tomography (micro-CT). Tissues were examined by H&E, Safranin-O, IHC, and IF staining. A flow diagram showing the process of establishing *Srgn*^−/−^ mice is presented in Fig. 3a. MRI was performed 8 weeks after AF puncture and analyzed according to the Pfirrmann MRI grading system. The T2-weighted signal intensities of wild-type (WT) or *Srgn*^−/−^ mice with AF puncture were stronger than those of WT mice with AF puncture (Fig. 3b, d). Micro-CT and disc height analyses showed that the disc heights notably decreased in the WT with AF puncture group compared to the WT group and increased in the *Srgn*^−/−^ plus AF puncture group compared to the WT plus AF puncture group (Fig. 3c, e). H&E, Safranin-O, and Sirius red (taken in bright field and polarized light) staining showed that the amount of NP tissue and disc height in the *Srgn*^−/−^ plus AF puncture group surpassed those in the WT plus AF puncture group and the histological scores of the WT plus AF puncture group were higher than those of the *Srgn*^−/−^ plus AF puncture group (Fig. 3f, h). IHC staining showed a significantly decreased percentage of COL2A1- and ACAN-positive cells, but the percentage of COL1A increased in the *Srgn*^−/−^ plus AF puncture group compared to the WT plus AF puncture group (Fig. 3g, j). In addition, IF staining and quantitative analyses showed that inhibition of SRGN significantly upregulated COL2A1 and ACAN expression, simultaneously diminishing COL1A expression in the *Srgn*^−/−^ plus AF puncture mice compared to that in the WT plus AF puncture mice (Fig. 3i, k). These results suggested that inhibition of SRGN could effectively alleviate the progression of IVDD.

### Late-stage NPCs with elevated SRGN aggravate the inflammatory responses

**Fig. 4:**
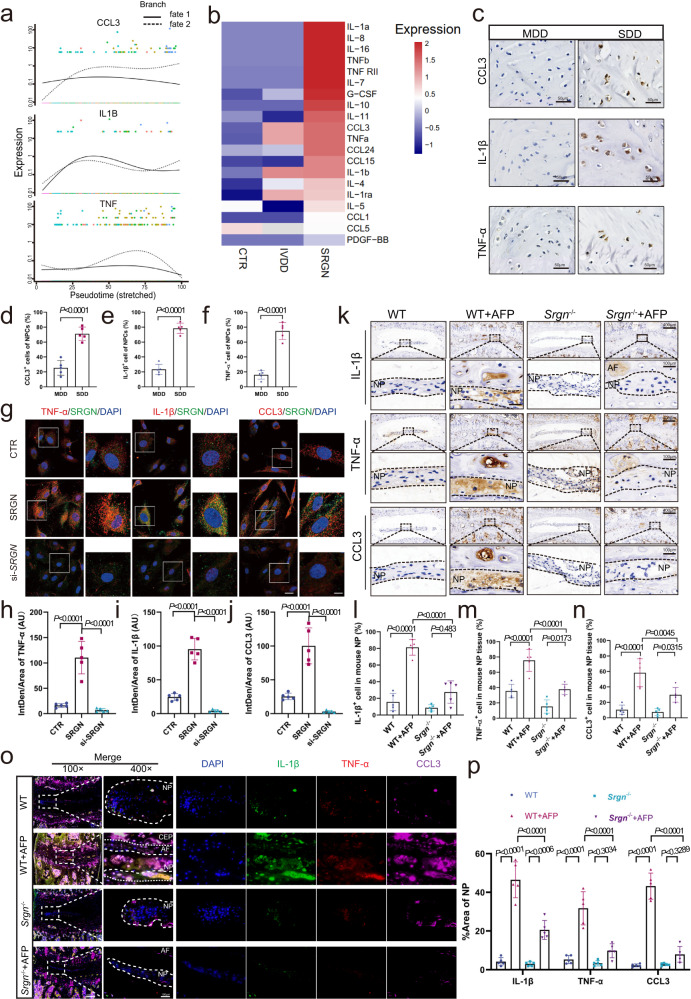
Late-stage NPCs with elevated SRGN aggravate the inflammatory responses. **a** Pseudotime kinetics of the *IL1B*, *TNF,* and *CCL3* genes. **b** Heatmap of significant inflammatory cytokines based on cytokine array results of different treatments of NPCs. **c**–**f** IHC staining and quantitative analysis of IL-1β, TNF-α, and CCL3 in MDD and SDD human IVDs (original magnification ×400, scale bar = 50 µm). **g**–**j** IF staining with quantitative analysis of IL-1β, TNF-α, and CCL3 in WT, *Srgn*^−/−^, WT plus AF, and *Srgn*^−/−^ plus AF mice 8 weeks after operation (original magnification ×100, ×400, scale bar = 400 µm, 100 µm). **k**–**n** IF staining of IL-1β, TNF-α, and CCL3 (red) co-stained with SRGN (green) in NPCs treated with SRGN or si-*SRGN* (original magnification ×400, ×1000, scale bar = 50 µm, 20 µm). **o**, **p** IF staining of IL-1β (green), TNF-α (red), and CCL3 (purple) in WT, *Srgn*^−/−^, WT plus AF, and *Srgn*^−/−^ plus AF mice (original magnification ×100, ×400, scale bar = 400 µm, 100 µm). CTR control group, IVDD 1% FBS starvation-induced NPC IVDD group, SRGN recombined SRGN-treated NPC group, SRGN siRNA-transfected NPC group, Intden/Area (AU) integrated density/area (arbitrary units). *n* = 5 each group. Data are represented as the mean ± standard deviation. *p* values were determined by two-tailed unpaired *t* tests for two-group comparisons and one-way ANOVA with a post hoc Bonferroni correction for multiple groups. Source data are provided as a Source Data file.

As described in previous studies, the local inflammatory response is one of the main causes of IVDD^24,25,39^. Therefore, we investigated the correlation between SRGN overexpression in late-stage NPCs and the local inflammatory response. First, pseudotime dynamic expression analysis of scRNA-seq data showed that *IL1B*, *TNF*, and *CCL3* were upregulated in late-stage NPCs (Fig. 4a). Then, quantitative measurement of 40 human inflammatory cytokines (Human Inflammation Array G3 [AAH-INF-G3], RayBiotech) was performed, and a heatmap revealed significantly elevated levels of inflammatory cytokines in the IVDD group (starvation-induced degeneration model, 1% FBS DMEM for 12 h) and SRGN group (treated with recombinant 1 μg/ml SRGN protein for 24 h) compared to the control group (Fig. 4b). IHC staining and quantitative analysis also showed augmented expression of IL-1β, TNF-α, and CCL3 in the SDD group compared to the MDD group (Fig. 4c–f). Furthermore, si-*SRGN* siRNAs were established and transfected into human NPCs (siRNA target sequences are provided in Supplementary Table 2). IF staining and quantitative analysis also revealed that inhibition of SRGN significantly downregulated the expression of IL-1β, TNF-α, and CCL3 (Fig. 4g–j).

In addition, IHC, IF staining, and quantitative analysis showed that the levels of IL-1β, TNF-α, and CCL3 were significantly decreased in the *Srgn*^−/−^ plus AF puncture mice compared to WT or WT plus AF puncture mice (Fig. 4k–n, o–p). These data indicated that SRGN played an essential role in the local inflammatory response in IVDD.

### SRGN in late-stage NPCs promotes inflammatory cytokine secretion

**Fig. 5:**
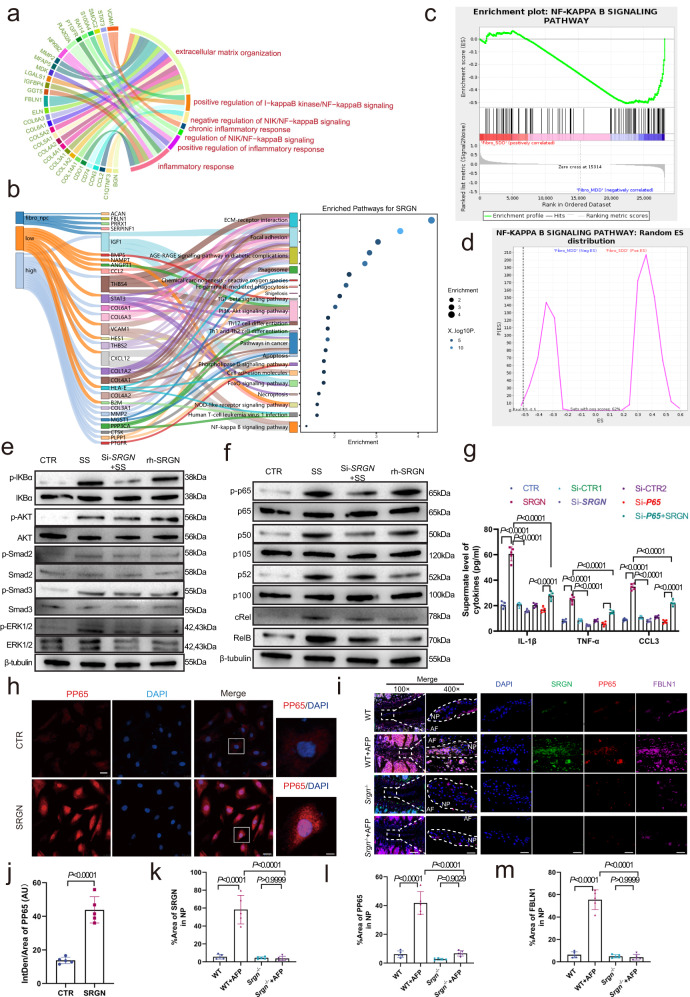
SRGN in late-stage NPCs promotes inflammatory cytokine secretion. **a**–**d** GO and pathway analysis and GSEA of scRNA-Seq in Fibro-NPCs. **e** Western blotting results of four candidate inflammatory signaling pathways ((the NF-κB, AKT, Smad2/3, and ERK1/2 pathways). **f** Western blotting screening results of candidate subunits of the NF-κB signaling pathway. **g** ELISA analysis of IL-1β, TNF-α, and CCL3 in NPCs transfected with si-*P65* and si-*SRGN*. **h**, **j** IF staining and quantitative analysis of pP65 (red) in human NPCs cultured with SRGN recombination protein (original magnification ×400, ×1000, scale bar = 50 µm, 20 µm). **i**–**m** IF staining of pP65 (red) costained with SRGN (green) and FBLN1 (purple) in WT, *Srgn*^−/−^, WT plus AF, and *Srgn*^−/−^ plus AF mice (original magnification ×100, ×400, scale bar = 400 µm, 100 µm). *n* = 3 each group in (**e**) and (**f**); *n* = 5 each group in (**g**–**m**). Data are represented as the mean ± standard deviation. *p* values were determined by a two-tailed unpaired *t* test for two-group comparisons and one-way ANOVA with a post hoc Bonferroni correction for multiple groups. Source data are provided as a Source Data file.

To further understand the mechanism by which SRGN regulates the local inflammatory response during IVDD, we conducted a KEGG analysis of the top 20 upregulated genes in SDD from the RNA-seq data, and the results indicated enrichment of the NF-κB signaling pathway (Supplementary Fig. 6c). KEGG analysis results of inflammatory cytokines array are shown in Supplementary Fig. 6d. In addition, GO and pathway analysis of single-cell Fibro-NPCs sequencing analysis are shown in Fig. 5a. Then, we set the *SRGN* expression level as an indicator and, in turn divided NPCs into two groups (*SRGN* expression above the median marked as ‘SRGN-Low’ and *SRGN* expression below the median marked as ‘SRGN-High’) to mimic *SRGN* knockdown and overexpression, respectively. Combining this information with the significant differentially expressed genes in late-stage NPCs (Fibro-NPCs) for KEGG analysis and pathway screening using Sankey diagrams, we found that the NF-κB signaling pathway was enriched (Fig. 5b). Gene set enrichment analysis (GSEA) results further indicated that late-stage NPCs were closely correlated with the NF-κB signaling pathway (Fig. 5c, d). In addition, by screening the 4 inflammation-related pathways above (the NF-κB, AKT, Smad2/3, and ERK1/2 pathways), Western blotting analysis revealed that the NF-κB signaling pathway was significantly activated, and phosphorylated P65 was significantly upregulated in the NPCs treated with recombinant SRGN protein (Fig. 5e, f). Enzyme-linked immunosorbent assays (ELISAs) analysis of NPCs showed that the expression levels of IL-1β, TNF-α, and CCL3 were decreased in NPCs transfected with si-*P65* and si-*SRGN*, which the administration of exogenous recombinant human SRGN could not reverse (Fig. 5g). IF staining also showed that the NF-κB signaling pathway was highly activated in SRGN-treated NPCs (Fig. 5h, j). Notably, IF staining results suggested augmented expression of pP65, SRGN, and FBLN1 (a marker of Fibro-NPCs) in WT plus AF puncture mice compared to WT or *Srgn*^−/−^ plus AF-puncture mice (Fig. 5i, k–m). These results indicated that the NF-κB signaling pathway was involved in SRGN-mediated regulation of the local inflammatory response in late-stage NPCs during IVDD.

### SRGN secreted by late-stage NPCs increases macrophage infiltration

**Fig. 6:**
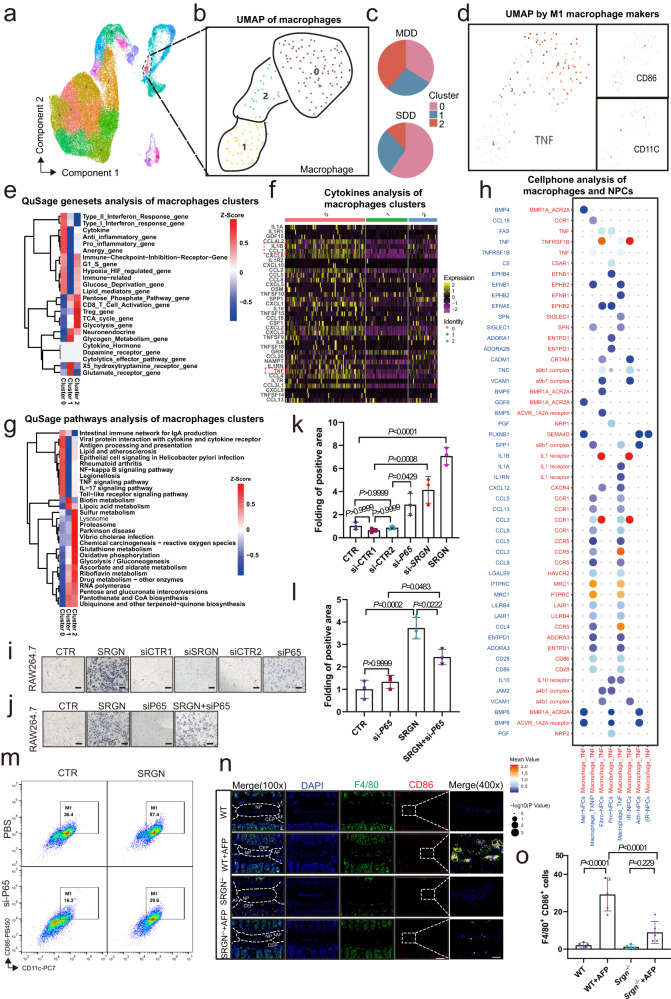
SRGN secreted by late-stage NPCs increases macrophage infiltration. **a**, **b** UMAP plot of the following 3 clusters of macrophages: cluster 0 (*TNF*^+^), cluster 1 (*VENTX*^+^), and cluster 2 (*MARCO*^+^). **c** The propositions of 3 clusters in MDD and SDD IVDs. **d** UMAP plot of cluster 0 (*TNF*^+^) macrophages and M1 macrophages. **e** QuSAGE gene set analysis heatmap showing that cytokine and proinflammatory gene sets are significantly expressed. **f** Seurat4RDSPlot analysis shows that *CCL3*, *IL1B*, and *TNF* are significantly increased in cluster 0 macrophages. **g** QuSAGE pathways analysis showed that the proinflammatory gene set and NF-κB signaling pathway gene set were significant in cluster 0 macrophages. **h** CellPhone and receptor analysis of *TNF*^+^ macrophages showed that *TNF*^+^ macrophages are regulated by Fibro-NPCs with CCL3, IL-1β, and TNF-α receptors. **i**–**l** Migration of macrophage RAW264.7 cells after SRGN, si-*SRGN* or si-*P65* treatment (original magnification ×100, scale bar = 200 µm). *n* = 3 each group. (m) Flow cytometry of CD11c+ and CD86 + RAW264.7 cells treated with SRGN and si-*P65*. **n**, **o** IF staining of F4/80 (green) and CD86 (red) and quantitative analysis in WT, *Srgn*^−/−^, WT plus AF, and *Srgn*^−/−^ plus AF mice (original magnification ×100, ×400, scale bar = 400 µm, 100 µm). *n* = 3 each group in (**i**–**m**), *n* = 5 each group in (**n**) and (**o**). Data are represented as the mean ± standard deviation. *p* values were determined by one-way ANOVA with a post hoc Bonferroni correction or a Kruskal‒Wallis *H* test with Dunn’s correction. Source data are provided as a Source Data file.

As described in previous studies, macrophages play an important role in IVDD^10,40^. To investigate the role of macrophages in regulating the local inflammatory response, macrophages were classified by gene expression (*CD16* and *CD68*) and functional analysis (Fig. 6a), and we generated a separate UMAP for macrophages. The macrophages were divided into the following 3 clusters: cluster 0 (*TNF*^+^ macrophages), cluster 1 (*VENTX*^+^ macrophages), and cluster 2 (*MARCO*^+^ macrophages) (Fig. 6b). A pie chart describing the proportions of the 3 clusters in MDD and SDD tissues suggested a higher proportion of *TNF*^+^ macrophages in the SDD group. In addition, *CD11c*^+^ and *CD86*^+^ cells, markers of M1 macrophages, were mainly distributed in *TNF*^+^ macrophages. Since CD11c^+^ CD86^+^ macrophages were defined as M1 macrophages, we assumed that *TNF*^+^ macrophages were a type of M1 macrophage that played an important role in IVDD (Fig. 6c, d). QuSage analysis of the 3 clusters revealed that the expression of inflammatory cytokines and proinflammatory gene sets was significantly upregulated in *TNF*^+^ macrophages (Fig. 6e). Moreover, the expression of the *CCL3*, *IL1B*, and *TNF* genes was strikingly elevated in the proinflammatory gene set in *TNF*^+^ macrophages (Fig. 6f).

Seurat4RDSPlot analysis was conducted for the 3 clusters, and heatmaps of the cytokine expression of the top 50 differentially expressed genes were created; the *CCL3*, *IL1B*, and *TNF* genes were highly expressed in *TNF*^+^ macrophages (Supplementary Fig. 7a). QuSage analysis showed that the NF-κB signaling pathway was highly enriched in *TNF*^+^ macrophages (Fig. 6g and Supplementary Fig. 7b). These results indicated that *TNF*^+^ macrophages (M1 polarized macrophages) regulated the local inflammatory response by modulating the NF-κB signaling pathway during IVDD.

Subsequently, to further investigate the correlation between *TNF*^+^ macrophages (M1 polarized macrophages) and the late-stage NPCs with elevated SRGN, CellPhoneDB analysis of *TNF*^+^ macrophages and NPC subclusters showed that the ligand and receptor pairs of TNF-α, CCL3 and IL-1β significantly increased in *TNF*^+^ macrophages compared to late-stage NPCs (Fibro-NPCs) (Fig. 6h). Transwell migration assays showed that the migration of RAW264.7 cells, classic macrophages commonly used to assess the function and mechanism of macrophages, was significantly increased in the SRGN-treated group, while the migration of RAW264.7 cells diminished in the si-*SRGN* and si-*P65* groups (Fig. 6i–k). Notably, flow cytometry analysis also suggested that treatment with recombinant SRGN protein (1 µg/ml, 24 h) increased the proportion of M1 polarized macrophages (CD11c+ and CD86+) in RAW264.7 cells, while treatment with si-P65 significantly reduced the proportion of M1 polarized macrophages induced by SRGN (Fig. 6m). Furthermore, IF and quantitative analysis showed that macrophage infiltration and M1 polarization increased in the WT plus AF puncture mice compared to the WT or *Srgn*^−/−^ plus AF puncture mice (Fig. 6n, o). These data indicated that *SRGN*-overexpressing late-stage NPCs aggravated the local inflammatory response by promoting macrophage infiltration and M1 polarization.

### DAP attenuates the local inflammatory response and alleviates IVDD

**Fig. 7:**
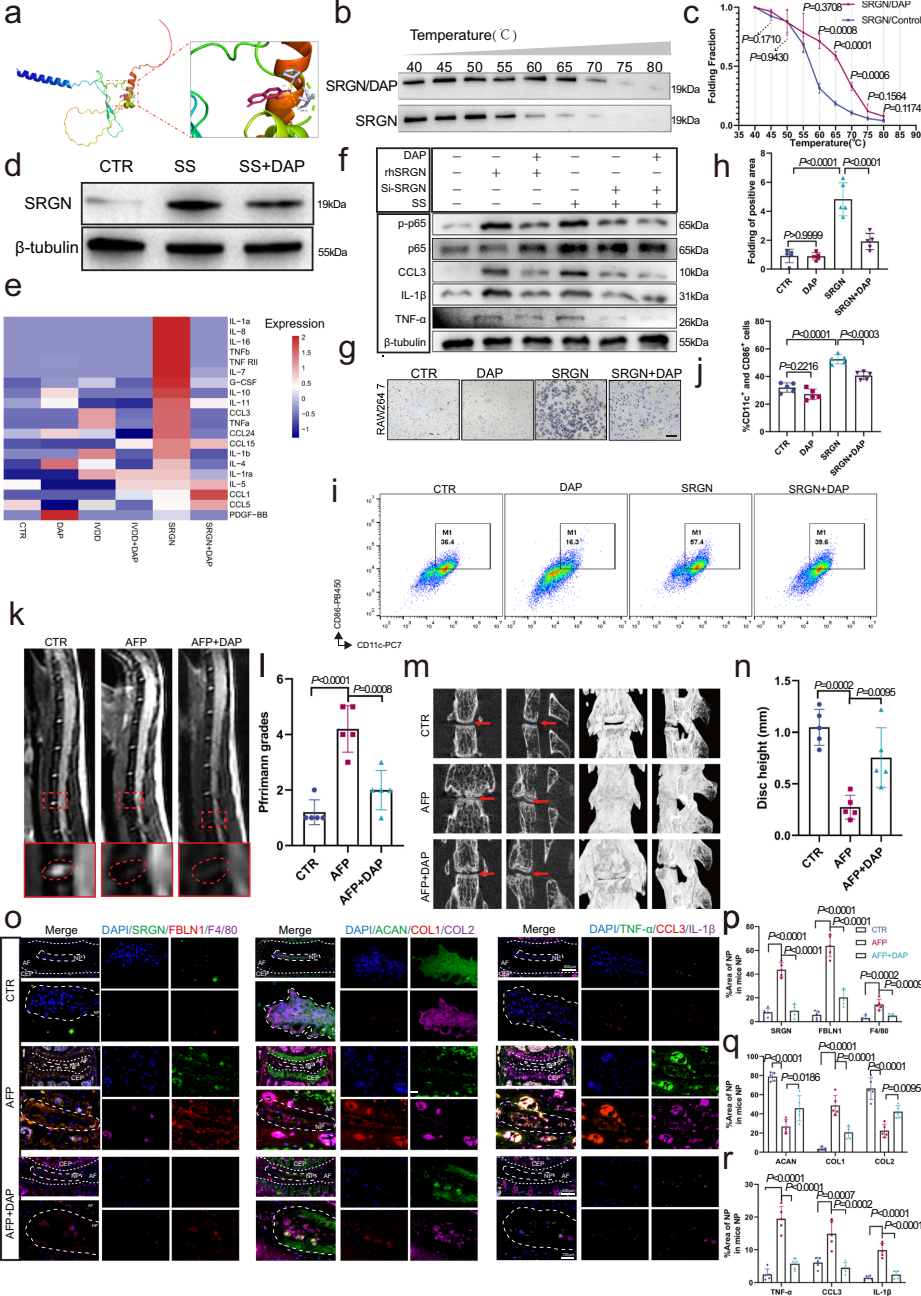
DAP attenuates the IVD local inflammatory response to alleviate IVDD. **a** Ligand interaction diagram of the top-scoring molecular docking complexes of the DAP and SRGN proteins (RMSD = 0.406; estimated free energy of binding = −15.313 kJ/mol). **b** CETSA of DAP and protein. **c** Principal criteria for evaluating the significance of protein stabilization in the melt curve. **d** Western blotting analysis of SRGN expression in the starvation-induced IVDD group and the DAP treatment group. **e** Heatmap of significant inflammatory cytokines based on cytokine array results of NPCs with different treatments. **f** Western blotting analysis of p-P65, CCL3, IL-1β, and TNF-α expression with different treatments. **g**, **h** Migration of RAW264.7 macrophages after si-*SRGN* or si-*P65* treatment (original magnification ×100, scale bar = 200 µm). **i**, **j** Flow cytometry of CD11c+ and CD86 + RAW264.7 cells treated with SRGN and DAP. **k**, **l** MRI images and Pfirrmann grade analysis of control, AF, and AF plus DAP mice 8 weeks after operation. **m**, **n** Micro-CT images and disc height analysis of control, AF, and AF plus DAP mice 8 weeks after operation. **o**–**r** IF staining of COL1A1, COL2A1, ACAN, IL-1β, TNF-α, CCL3, and F4/80 in AF and AF plus DAP mice 8 weeks after operation (original magnification ×100, ×400, scale bar = 400 µm, 100 µm). CTR control group, SS serum starvation; *n* = 3 each group in (**b**–**d**) and (**f**), *n* = 5 each group in (**g**–**r**). Data are represented as the mean ± standard deviation. *p* values were determined by one-way ANOVA with a post hoc Bonferroni correction. Source data are provided as a Source Data file.

Next, we investigated whether a drug or compound could effectively alleviate IVDD by regulating the local inflammatory response. First, in the absence of experimental structures, AlphaFold2 software was used to predict the SRGN structure and assess its reliability (Fig. S8a–d). Then, docking prediction analysis showed that DAP could effectively bind to SRGN (Fig. 7a; RMSD = 0.406; binding energy = −15.313 kJ/mol). A cellular thermal shift assay (CETSA) of DAP and SRGN proteins also suggested that DAP could bind to SRGN in vivo (Fig. 7b, c).

We evaluated DAP toxicity in NPCs and chose the optimum concentration (2 μM) and duration for the following experiments (Supplementary Fig. 8e and f). Western blotting analysis showed that DAP downregulated the expression of SRGN in NPCs subjected to starvation conditions (Fig. 7d). The heatmap of inflammatory cytokines and Western blotting results suggested that DAP significantly decreased the expression of *TNF*, *CCL3*, and *IL-1β* in NPCs treated with recombinant SRGN protein compared to NPCs treated with SRGN alone (Fig. 7e, Supplementary Fig. 8g). Western blotting analysis also indicated that DAP substantially diminished the level of p-p65 in NPCs treated with recombinant SRGN protein. However, the inhibitory effect of DAP on p-p65 was limited after SRGN expression was knocked down (Fig. 7f). Furthermore, the migration assay of RAW264.7 cells showed that DAP administration markedly reduced the migration of RAW264.7 cells (Fig. 7g, h). Flow cytometry analysis suggested that the proportion of M1 macrophages notably decreased in RAW264.7 cells treated with DAP (2 μM, 24 h) plus SRGN compared to RAW264.7 cells treated with SRGN alone (Fig. 7i, j). In addition, MRI and Pfirrmann grading analysis showed that DAP augmented the T2-weighted signal intensities in the AF plus DAP group compared to those in the AF group (Fig. 7k, l). Micro-CT and disc height analysis suggested that DAP helped maintain the disc height in AF plus DAP mice compared to AF mice (Fig. 7m, n). H&E and Safranin-O staining showed that the amount of NP tissue and the disc height were preserved in the AF puncture plus DAP group compared with that in the AF puncture group. IHC staining and quantitative analysis results presented a higher percentage of COL1A1-, IL-1β-, TNF-α-, CCL3-, and F4/80-positive cells and a lower percentage of COL2A1- and ACAN-positive cells in AF mice than in AF plus DAP mice (Fig. S7h–k). Furthermore, the IF staining and quantitative analysis results were similar to the IHC results (Fig. 7o–r). Together, these results suggested that DAP attenuated the local inflammatory response to alleviate IVDD by downregulating the expression of SRGN in vivo.

## Discussion

In this study, we aimed to investigate the role of specific cell subpopulations of NPCs in IVDD and explore potential therapeutic strategies. In degenerative NPCs, especially late-stage NPCs, upregulation of SRGN promoted the local inflammatory response by elevating the secretion of inflammatory cytokines and promoting macrophage infiltration and M1 polarization, demonstrating a mechanism of IVDD. Furthermore, the potential therapeutic effect of DAP in alleviating the progression of IVDD by suppressing SRGN was studied both in vitro and in vivo.

LBP is a leading cause of disability, and the focus of research has been early stage diagnosis and identification of effective therapies for middle- and late-stage IVDD^41^. Many studies support the idea that cell-based strategies could be used to effectively diagnose and alleviate IVDD. Previous studies have confirmed that changes in NPCs play a prominent role in IVDD, but it is still unclear which NPC type matters. In our scRNA-seq results (MDD: *n* = 4, two males and two females; SDD: *n* = 3; one male and two females; age 41.14 ± 18.01 years), the NPCs were divided into six subclusters based on marker genes and characteristics of NPCs. Furthermore, Fibro-NPC NPCs were found to exist at the end of the pseudotime trajectory. They were mainly distributed in degenerative NP tissues, and were therefore defined as late-stage NPCs. Our results suggest that late-stage NPCs are an effective cell-based marker for IVDD. Subsequently, to explore gene divergence and identify potential biomarkers for late-stage NPCs, we conducted scRNA-seq analysis, which confirmed that SRGN expression was significantly augmented in SDD tissue compared with MDD tissue, especially in late-stage NPCs. We also found differential expression of SRGN in mild and severe degenerative IVDs. Notably, SRGN, which is typically expressed at low levels in normal IVDs and predominantly found in liver, lung, and fat tissue, emerged as a potential biomarker for degenerative NPCs, and particularly late-stage NPCs. SRGN could serve as a preclinical diagnostic marker and a potential therapeutic target for IVDD.

To further identify the role of SRGN-upregulated late-stage NPCs in IVDD, we established an IVDD mouse model combined with an SRGN knockout mouse model^24,25,42^. MRI and CT are the most commonly used techniques to determine the status of IVDs in patients. Similarly, micro-CT is widely used to measure disc height and osteophytes as parameters of IVDD in small animals^43,44^. We previously reported that MRI can be used to measure IVDD in rats; however, performing MRI on mice is difficult due to their small body size, and IVDs are hard to visualize clearly. In this study, we used specially made coils and successfully showed that the IVDD grade was significantly alleviated in the *SRGN* knockout group. Based on the micro-CT scan and IHC staining results, IVDD was considerably alleviated in the *Srgn*^−/−^ mice, indicating that SRGN plays an important role in the IVDD process.

The precise mechanism by which SRGN-elevated late-stage NPCs aggravate IVDD is unknown. IVDD is a multifactorial disease with an increased local inflammatory response in the IVD^24,25^. Recent studies have suggested that SRGN is involved in the inflammatory response^45–47^. Angela et al. and Michele et al.^18,20^. suggested that SRGN overexpression with IL-1β or LPS could induce an inflammatory response in chondrocytes. Our results revealed that SRGN-elevated late-stage NPCs overexpressed many inflammatory cytokines in NP tissues, so we hypothesized that these late-stage NPCs played an essential role in the local inflammatory response to aggravate IVDD. According to our previous studies, TNF-α, IL-1β, and CCL3 are the most important cytokines and chemokines expressed during IVDD, and they initiate and amplify inflammation through different pathways. Our results also found that inflammatory cytokines were upregulated in IVDD. A previous study confirmed that SRGN plays an important role in the storage and secretion of many cytokines, chemokines, and proteases and is thus involved in many physiological and pathological processes^16^. Interestingly, our results suggested that the expression of inflammatory cytokines was significantly downregulated in SRGN knockout mice, even in IVDD model mice. According to these data, the downregulation of SRGN significantly mitigated the local inflammatory response of IVD. Together, these results verified our hypothesis that SRGN plays a crucial role in the process of IVDD by regulating the local inflammatory response of IVD. This observation suggests that elevated SRGN may serve as a preclinical diagnostic marker and an important therapeutic target for IVDD.

The mechanisms by which SRGN-elevated late-stage NPCs exacerbate the local inflammatory response of IVDs to aggravate IVDD remain unclear. Previous studies have demonstrated that SRGN regulates several signaling pathways in various diseases. For example, SRGN was shown to regulate TGF-β2 signaling in breast cancer, activate the NF-κB pathway in non-small lung cancer, participate in mitogen-activated protein kinase (MAPK)/β-catenin signaling in nasopharyngeal cancer and regulate the PI3K, Rac, and Src signaling pathways in breast cancer^48–51^. The NF-κB signaling pathway is a classic signaling pathway that regulates the inflammatory response^52^. With the rapid development of bioinformatics analysis, an increasing number of basic scientific studies have been performed to effectively assess differential gene expression and predict a gene’s up/downstream targets and signaling pathways. In this study, our findings indicated that SRGN could activate the NF-κB signaling pathway by increasing the phosphorylation of P65 and that SRGN promoted the secretion of inflammatory cytokines to exacerbate the local inflammatory response in IVDs by activating the NF-κB signaling pathway, thereby aggravating IVDD. These findings further confirmed that SRGN-elevated late-stage NPCs promoted inflammatory cytokine secretion to exacerbate the local inflammatory response via activating the NF-κB signaling pathway in IVDD.

Moreover, an interesting phenomenon was found: macrophage infiltration was significantly increased in degenerative NP tissues. This finding suggested that SRGN-secreting late-stage NPCs might induce macrophage infiltration. Recent studies have shown that M1 polarization, chemotaxis, and inflammatory infiltration of macrophages also play important roles in IVDD^10,53^. In our scRNA-Seq results, *TNF*^+^ macrophages (cluster 0 in macrophages) were mainly distributed in the SDD group. Further scRNA-Seq analysis indicated an overt correlation between the inflammatory response and *TNF*^+^ macrophages, where NF-κB signaling was also strongly activated. Results of the chemotaxis assay of macrophages also showed that *SRGN* played an important role in *TNF*^+^ macrophage infiltration and M1 polarization. These results suggested that SRGN-secreting late-stage NPCs were also involved in the exacerbation of the local inflammatory response in IVDs by chemotactic macrophages. Together, these results identify a mechanism of IVDD, which may contribute to developing diagnostic and therapeutic strategies.

As most late-stage IVDD-related spinal diseases require surgical treatment, it is meaningful to prevent IVDD at early stages and to seek natural products, drugs, or compounds that can directly or indirectly suppress SRGN expression to alleviate IVDD^54^. We used AlphaFold2 software to predict the SRGN structure. According to its protein structure, we further assessed its binding affinity with various compounds. Among them, DAP exhibited the highest potential for binding with SRGN. DAP is a natural coumarin derivative extracted from plants of the genus *Daphne* and has been reported to exhibit various pharmacological activities, particularly anti-inflammatory properties^26^. We revealed that DAP downregulated SRGN expression to decrease the secretion of inflammatory cytokines and macrophage infiltration, mitigating the progression of IVDD. DAP has been synthesized and incorporated into drugs for clinical practice such as treatment of thromboangiitis obliterans and other occlusive vascular diseases^31^. These findings indicate that DAP could become an effective therapeutic strategy for IVDD based on its ability to suppress SRGN expression and alleviate the local IVD inflammatory response.

Despite these important findings, there are some limitations in this study. First, the *SRGN* global knockout mice we used had a deficiency in that we could not exclude the role of other cells in discs such as immune cells; thus, elevated levels of SRGN can only serve as a potential biomarker of IVDD rather than NPCs. Second, normal and severe IVDD (Grade V) samples of human IVDs are difficult to obtain. Although the MDD NP samples used in this study were close to normal, gene expression in these samples may still differ from that in completely normal healthy human NP samples. Third, due to the limitation of small animal MRI, there was no precise grading system for the IVDD in the mouse model. Thus it was difficult to distinguish mild from severe degeneration, so we divided the mouse models into control and IVDD groups. Finally, although the IVDD animal model used is well accepted, the triggers in rodent models may not be identical to those in humans. Finally, we did not perform a high-throughput screen of a library of compounds; computational predictions are not equal to experimental structures, but could provide valuable insights and hypotheses for further investigation.

In summary, we report that late-stage NPCs with upregulated SRGN expression aggravate the pathogenesis of IVDD. Subsequently, we confirmed that SRGN could augment the secretion of inflammatory cytokines and increase macrophage infiltration to aggravate IVDD. Then, we demonstrated that DAP effectively downregulated the expression of SRGN, mitigating the local inflammatory response of IVDs and alleviating IVDD. Therefore, this study highlights that SRGN was upregulated in degenerative NPCs, especially in late-stage NPCs, and it may become a preclinical diagnostic biomarker and therapeutic target for IVDD. Furthermore, we found that DAP could be a treatment option for IVDD.

## Methods

### Ethics statement

The use of patient samples was approved by the Ethics Committee of The First Affiliated Hospital of Sun Yat-sen University. Protocol of animal model of intradiscal injection was approved by The Institutional Animal Care and Use Committee (IACUC) at The First Affiliated Hospital of Sun Yat-sen University (No. [2020]017). The study was performed in accordance with the Declaration of Helsinki. The study was approved by China’s Ministry of Science and Technology related to the export of genetic information. Informed consent was obtained from all patients involved in the study, sample was obtained in necessary operations and patients received no compensation in this study.

### Human IVDs with different grades of degeneration

Between September 2016 and June 2021, we collected 48 IVD samples from patients (19 females and 29 males, age 47.81 ± 21.87 years). The degree of disc degeneration was evaluated according to the Pfirrmann grading system. Normal IVDs were obtained from patients who had experienced trauma, and degenerated IVDs were obtained from patients with degenerative spinal diseases (disc herniation, spinal canal stenosis, and degenerative scoliosis).

### In vitro culture of NPCs

Eight human NP tissue specimens were used for isolation and culture of NPCs using the method described in our previous study^39^. Cells were cultured in DMEM (Invitrogen, CA, USA) containing 10% FBS (Invitrogen, CA) and antibiotics (Invitrogen, CA), in a humidified incubator containing 5% CO_2_ at 37 °C. The cells were harvested using a solution containing trypsin (0.25%) and EDTA (1 mM) (Invitrogen, CA), and were then subcultured in 10-cm dishes.

The NPCs were seeded in 6-well plates, grown to 80% confluency, and treated with 2 µM DAP (Abcam, ab143113, USA) or 1 µg/ml SRGN (R&D, 10190-SN-050, USA) for 24 h for use in subsequent experiments. Furthermore, NP cells were cultured in 1% FBS medium for 12 h to establish a starvation-induced IVD NP degeneration model.

### Murine IVDD model

A murine IVDD model was established via surgery performed under aseptic conditions as described in our previous study^24,39^. C57BL/6 mice weighing ~30 g and that were 8–9 weeks old (*n* = 5 per group) were raised in SPF environment under constant temperature (23–25 °C) and humidity (50%) with a 12-h light/12-h dark circadian cycle. For surgery, the mice were placed in the prone position, and a midline longitudinal incision was made on their back. The left facet joint between the third and fourth lumbar vertebrae was removed while visualizing the L3/4 IVD. A 26-gauge needle was inserted into the disc 1.0 mm parallel to the endplates for 30 s (AF puncture group). A clamp was used as a depth stop to prevent injury to the adjacent tissue. The muscles were then closed using 3-0 silk suture, and the skin was closed with using 4-0 nylon suture.

DAP was diluted to 3 mg/ml with normal saline. The mice in the DAP plus AF puncture group were intraperitoneally injected with DAP at various concentrations (0, 1, 3, or 5 mg/kg/week), and 3 mg/ml resulted in the greatest improvement in IVDD. One week after the operation, 3 mg/kg/week of DAP was intraperitoneally injected into the mice for 3 consecutive weeks. Mice in the control group received no treatment following surgery.

### Srgn^−/−^ mice

Animal study was conducted by 7–8 weeks *Srgn* knockout C57BL/6 (*n* = 5 per group) which were generated using CRISPR/Cas9-mediated genome editing technology by Cyagen (Suzhou, China). The gRNA for the mouse *SRGN* gene and Cas9 mRNA were coinjected into fertilized mouse eggs to generate targeted knockout offspring. F2 founder animals were identified by PCR (Supplementary Fig. 5a–f), followed by sequencing analysis. Heterozygous mice were then bred to assess germline transmission and F3 animal generation. IHC staining of SRGN was performed in WT and *Srgn*^−/−^ IVD tissue (Supplementary Fig. 6a). All mice were raised in SPF environment under constant temperature (23–25 °C) and humidity (50%) with a 12-h light/12-h dark circadian cycle.

### Micro-computed tomography (CT) and magnetic resonance imaging (MRI)

At 8 weeks after surgery, micro-CT and MRI were performed on all mice before sacrifice. After anesthetization, the mice were placed in the prone position with their spine straight. NRecon, Dataviewer, and CTvox were used to reconstruct and create three-dimensional images of the micro-CT scans. The degree of degeneration on MRI was determined according to the Pfirrmann grade. The IVD height was measured using the method presented in the Materials section (Supplementary Fig. 4g).

### Western blotting analysis

The protein of treated NPCs was extracted and electrophoretically separated with 10% or 15% SDS-PAGE. Subsequently, membranes were blocked with 3% bovine serum albumin (BSA) and incubated with primary antibodies. The primary antibodies included anti-SRGN (1:1000, SAB2103016, Sigma-Aldrich; 1:1000, ab156991, Abcam), anti-IL-1β (1:1000, ab254360, Abcam), anti-TNF-α (1:1000, ab183218, Abcam), anti-CCL3 (1:1000, ab259372, Abcam), and anti-β-actin (1:1000, ab8226, Abcam) (for more antibody information see Supplementary Table 3). After washing with PBS, the membranes were incubated with the following secondary antibodies: anti-rabbit IgG (1:5000, 7074, Cell Signaling Technology) or anti-mouse IgG (1:5000, 7076, Cell Signaling Technology). Finally, Western blotting bands were detected using enhanced chemiluminescence detection reagents (Invitrogen, CA, USA) (uncropped gel image were supplied in Supplementary Fig. 11).

### Enzyme-linked immunosorbent assay (ELISA)

NPCs were grown to confluence in six-well plates, and then treated as needed for 24 h in an incubator at 37 °C with 5% v/v CO_2_. Cell supernatants were collected and analyzed for TNF-α, IL-1β, or CCL3 using specific ELISA kits (DTA00D, DLB506, DMA00, R&D Systems), according to the manufacturer’s protocol.

### Immunohistochemistry (IHC) and histopathological analysis

Tissue specimens were embedded in paraffin and cut into 5-µm sections. Subsequently, the sections were deparaffinized and rehydrated, followed by H&E and Safranin-O staining, or antigen retrieval with 0.01 M sodium citrate. The sections were blocked with 3% hydrogen peroxide and 5% normal goat serum. The slides were incubated with the following primary antibodies: anti-SRGN (1:200, HPA000759, Sigma-Aldrich), anti-CCL3 (1:200, ab259372, Abcam), anti-IL-1β (1:200, ab283818, Abcam), anti-collagen II (1:1000, 13141, Cell Signaling Technology), and Aggrecan (1:200, 3033, Cell Signaling Technology) (more antibody information see Supplementary Table 3). The sections were incubated with a secondary antibody, and then developed with DAB solution. Hematoxylin was used for nuclear staining. Finally, the sections were observed and imaged under an Olympus BX63 microscope at ×10, ×50, and ×400 magnifications, and the percentages of SRGN^+^, IL-1β^+^, CCL3^+^, collagen II^+^, and aggrecan^+^ cells in the IVD samples were quantified using ImageJ software (National Institutes of Health, Bethesda, MD, USA). The histologic scores were then assessed^24^: normal discs(5), moderately degenerated disc(6-11), and severely degenerated disc(12-14).

### Immunofluorescence analysis

NPCs were grown on confocal plates, incubated for 24 h and then treated as needed for 24 h. Next, the cells were fixed for 15 min with 4% formalin, and then permeabilized for 10 min with 0.1% Triton X-100. After washing, cells were blocked for 1 h with 10% goat serum. IF analysis of IVD tissue was similarly to that of cells after fixation. Briefly, the disc tissues embedded in paraffin were sectioned and deparaffinized in xylene, followed by dehydration in isopropanol dilutions. Antigen retrieval was performed by heating and blocking with 10% goat serum for 1–2 h. Cells/tissues were then incubated with antibodies against TNF-α (1:200), IL-1β (1:200), CCL3 (1:500) F4/80(1:100), CD86(1:100) and pp65(1:100) (for more antibody information see Supplementary Table 3). The samples were washed 3 times with 0.1% PBST, and then incubated with anti-mouse/rabbit Alexa 488/555/594-conjugated secondary antibody (1:2000, A-11008/A-10680/A-21429, Thermo Fisher Scientific) for 2 h at room temperature. The samples were washed again, and the DNA was stained with DAPI (6 μl DAPI in 100 μl PBS) for 0.5 h at room temperature. Wash samples and digital images were obtained using a confocal laser scanning microscope (Leica), according to the manufacturer’s instructions.

### Molecular docking analysis

#### Structure prediction

The structure of SRGN was predicted using AlphaFold 2. The model quality was assessed using the predicted aligned error, in which the color at position (x, y) indicated the expected position error at residue x when the predicted and true structures were aligned on residue y (Fig. S8b and c).

#### Molecular docking and visualization

The DAP structure was obtained from the Zinc database (Fig. S5a). The molecular dockings of the predicted SRGN and DAP structures were simulated and analyzed with AutoDockTools-1.5.6, and visualized with PyMOL 2.3.2.

### Cellular thermal shift assay (CETSA)

NPCs were pretreated with DAP for 12 h, and then collected and washed with PBS. Next, 2 × 10^5^ cells per ml were resuspended in PBS containing 1% protease inhibitor cocktail. The cells were divided into equal volumes, heated at the indicated temperatures for 3 min, and cooled to 20 °C for 3 min. Cells were lysed by 3 freeze-thaw cycles in liquid nitrogen. After each freeze-thaw cycle, the lysate was briefly vortexed to ensure homogenous thawing. The treated samples were centrifuged at 16.2 × *g* for 15 min at 4 °C. For removal of the insoluble proteins, the soluble fractions were subjected to SDS-PAGE Western blotting and quantitative analysis.

### Macrophage migration assay

In vitro cell migration assays were performed in 12-mm diameter and 8-μm-pore polycarbonate filter Transwell plates (Corning Transwell polycarbonate membrane cell culture inserts). The RAW264.7 cells used in this study were obtained from Sunncell Biotech (Cat#. SNL-112, Wuhan, China). Cell line STR authentication report was provided in Supplementary Table 5. RAW264.7 cells (2 × 10^5^ cells in 200 μl 1% FBS DMEM) were seeded in the upper chamber, and the lower well was filled with treated NPCs. After incubation for 16–18 h at 37 °C in the presence of 5% CO_2_, the RAW264.7 cells were fixed for 15 min in methanol and stained for 5–10 min with 0.1% crystal violet. Cells that had migrated to the bottom surface of the filter were counted.

### In vitro small interfering RNA (siRNA) transfection

SiRNAs (sequence information is available in Supplementary Table 2) were constructed by RiboBio (Guangzhou, China) and used to inhibit the expression of *SRGN* and *P65*. Human NP cells were cultured in six-well plates to 60–70% confluence and were transfected with 50 nM negative control (si-CTR), *SRGN* or *P65* siRNA using Lipofectamine 3000 (Invitrogen) according to the manufacturer’s instructions. After 48 h, cellular lysates were obtained to analyze the expression of the genes of interest.

### Flow cytometry

The treated RAW264.7 cells were concentrated, and washed with PBS twice. After washing, cells were incubated with fluorescence-labeled surface antibodies against CD11c and CD86 (eBioscience) for 30 min at 4 °C (for antibody information see Supplementary Table 3). Fluorescence signals were detected using the Beckman CytoFLEX Flow Cytometer.

### RNA sequencing and analysis

Total RNA was extracted from NP tissue, and treated with the GeneRead™ rRNA Depletion kit (Qiagen, Hilden, Germany, Cat No. 180211) to remove ribosomal RNA. Then, a VAHTS Stranded RNA-seq Library preparation kit for Illumina (Vazyme, Nanjing, China, Cat) was used to construct strand-specific libraries. Sequence reads were then aligned to the human genome (version Hg38) using HISAT2 (version 2 2.1.0) and assessed. After alignment, Htseq (Version 0.11.0) was used to calculate the read count mapped to the genome. The expression data were standardized with fragments per kilobase million reads (FPKM) to facilitate comparison of gene expression levels between groups. The DESeq2 (1.22.2) algorithm was used to filter the differentially expressed genes after significance and false discovery rate (FDR) analysis, and the criteria were as follows: (i) log2FC > 1 and (ii) FDR < 0.05. Based on the differentially expressed gene analysis, a volcano plot was drawn with R software using different colors. Gene ontology (GO) and pathway enrichment analysis were performed using DAVID (https://david.ncifcrf.gov/, Version 6.8).

### Single-cell RNA sequencing

scRNA-seq data analysis was performed by NovelBio Bio-Pharm Technology Co., Ltd. with NovelBrain Cloud Analysis Platform. We applied fastp with default parameter filtering of the adaptor sequence to remove the low-quality reads and obtain the clean data. Then the feature-barcode matrices were obtained by aligning reads to the human genome (GRCh38 Ensemble: version 104) using CellRanger v6.1.1. We performed the down sample analysis of samples sequenced according to the mapped barcoded reads per cell of each sample and finally created the aggregated matrix. Cells containing over 200 expressed genes and a mitochondrial UMI rate below 20% passed the cell quality filtering and mitochondrial genes were removed from the expression table.

Seurat package (version: 4.0.3, https://satijalab.org/seurat/) was used for cell normalization and regression based on the expression table according to the UMI counts of each sample and percent of mitochondria rate to obtain the scaled data. PCA was constructed based on the scaled data with the top 2000 highly variable genes, and the top 10 principals were used for UMAP construction (resolution: 0.8). Utilizing graph-based cluster method, we acquired the unsupervised cell cluster result based on the top 10 principal-components of PCA, and we calculated the marker genes by FindAllMarkers function with the Wilcox rank sum test algorithm under the following criteria:1. log2FC > 0.25; 2. *p* value < 0.05; 3. min.pct>0.1. To identify the cell type in detail, the clusters of the same cell type were selected for re-UMAP analysis, graph-based clustering, and marker analysis.

Differential Gene Expression Analysis was performed as follows**:** To identify differentially expressed genes among samples, the function FindMarkers with the Wilcox rank sum test algorithm was used under the following criteria:1. log2FC > 0.25; 2. *p* value < 0.05; 3. min.pct>0.1.

GO term and pathway enrichment analysis was performed as follows: To study functional annotation and functional enrichment of markergenes and differentially expressed genes, GO term and pathway enrichment analysis was used under following criteria: Log2FC > 0.1 or Log2FC < −0.1.

Cell communication analysis was performed as follows: for a systematic analysis of cell–cell communication molecules, we applied cell communication analysis based on the CellPhoneDB (version: v1.1.0), a public repository of ligands, receptors and their interactions. Membrane, secreted and peripheral proteins of the cluster of different time point was annotated. significant mean and cell communication significance (*p* value < 0.05) were calculated based on the interaction and the normalized cell matrix achieved by Seurat Normalization.

QuSAGE analysis (gene enrichment analysis) was performed as follows: to characterize the relative activation of a given gene set such as pathway activation we performed QuSAGE (2.16.1) analysis. The scaled values of statistically significant normalized enrichment scores (Z scores) calculated by QuSAGE.

ssGSEA (single-sample gene set enrichment analysis) was performed as follows: to identify NPCs biological function, we applied the ssGSEA function of the GSVA package based on the NPCs gen set, which was constructed based on the differentially expressed genes of the NP tissues.

To predict the relative differentiation state of cells, we performed Cytotrace (v0.1.0) analysis.

Pseudo-time analysis: We applied the single-Cell trajectory analysis utilizing Monocle2 (http://cole-trapnell-lab.github.io/monocle-release) using DDR-Tree and default parameters. Before Monocle analysis, we selected marker genes of the Seurat clustering result and raw expression counts of the cell passed filtering. Based on the pseudo-time analysis, branch expression analysis modeling (BEAM Analysis) was applied for branch fate determined gene analysis.

### Cytokine array

Two hundred microliters of undiluted medium was applied to the Human Quantibody® Human Inflammation Array 3 (RayBio, Norcross, GA) according to the manufacturer’s instructions. This array concurrently detected and processed 40 human cytokines. First, after the glass slide was completely air dried, the array chips were incubated with sample diluents for 2 h at room temperature to act as a block. Undiluted medium (100 μl) was then added to the wells of the array and incubated for 2 h at room temperature. A standard cytokine dilution was added to the wells of the array to determine protein concentrations. For signal detection, 80 μl of Cy3-streptavidin was added to each well, and the array was rinsed and visualized by laser scanner. The RayBio® Analysis Tool (RayBio®, Norcross, GA) was used for protein classification. The flow diagram for cytokine array is shown in Supplementary Fig. 9, and raw data are listed in the Source Data file (raw data of the cytokines array).

### Statistical analysis

The data are presented as mean ± standard deviation. The normality of the data distribution was evaluated using the Shapiro‒Wilk test, and the distinctions between the two groups were analyzed using the *t* test or Wilcoxon rank-sum test, as deemed suitable. For multiple group comparisons, the appropriate statistical approach involved ANOVA with a Bonferroni correction or the Kruskal-Wallis H test with a Dunn’s correction. All statistical analyses were performed using SPSS software version 26.0 for Windows (IL, USA). A value of *P* < 0.05 (*P*-value adjustment in multiple comparison was performed using Bonferroni correction or Dunn’s correction) was considered statistically significant. Reported results were consistently replicated across multiple experiments with all replicates generating similar results: cell experiments: 3 independent experiments, animal experiments: 5 animals per group.

### Reporting summary

Further information on research design is available in the Nature Portfolio Reporting Summary linked to this article.

### Supplementary information

Supplementary Information

Reporting Summary

### Source data

Source Data

## Data Availability

All data are available in the main text or the materials. The raw sequence data reported in this paper have been deposited in the GEO database under accession code GSE244889. The data supporting the findings of this study are available from the corresponding authors upon reasonable request. Source data are provided with this paper.
