# Supplementary material for: Serglycin secreted by late-stage nucleus pulposus cells is a biomarker of intervertebral disc degeneration
Source: Nat Commun. 2024 Jan 2;15:47. doi: 10.1038/s41467-023-44313-9 (PMC10761730; doi:10.1038/s41467-023-44313-9)
Supplement: Supplementary file 1 — Supplementary Information [file 41467_2023_44313_MOESM1_ESM.pdf]

Supplementary Information on

Serglycin secreted by late-stage nucleus pulposus cells is a biomarker of intervertebral disc degeneration

Fan Chen [1, 2, 3†]; Linchuan Lei[1, 2, 3†]; Shunlun Chen[1, 2†]; Zhuoyang Zhao[1, 2, 3†]; Yuming Huang[1, 2]; Guowei Jiang [1, 2, 3]; Xingyu Guo[1, 2]; Zemin Li[1, 2;] Zhaomin Zheng [1, 2] *; and Jianru Wang [1, 2] *

[1]Department of Spine Surgery, The First Affiliated Hospital of Sun Yat-sen University, Guangzhou 510080, P.R. China

[2]Guangdong Province Key Laboratory of Orthopaedics and Traumatology, Guangzhou, 510080, P.R. China

[3]Laboratory of General Surgery, The First Affiliated Hospital, Sun Yat-sen University, Guangzhou 510080, China

[†]These four authors contributed equally to this work.

*Corresponding author:

Jianru Wang

Email: wangjru@mail.sysu.edu.cn

Telephone: +8613560182502

Fax: +862087332150

Address: No. 58, Zhongshan 2nd Road, Guangzhou, China 510080

Zhaomin Zheng

Email: zhzhaom@mail.sysu.edu.cn

Telephone: +8613925187872

Fax: +862087332150

Address: No. 58, Zhongshan 2nd Road, Guangzhou, China 510080

Supplementary Tables

Supplementary Table 1. The number and ratios % of cells sequenced for each patient and the each cell type

Clusters	Samples							Sum	Proposition (%)	Celltype
	**1**	**2**	**3**	**4**	**5**	**6**	**7**			
0	1144	295	1951	2033	822	387	134	6766	12.67	NP
1	581	480	780	847	1094	933	1030	5745	10.76	NP
2	53	191	188	20	113	1151	3699	5415	10.14	NP
3	714	51	1557	1968	73	266	176	4805	9.00	NP
4	77	88	345	151	2188	983	222	4054	7.59	NP
5	2026	82	511	1280	52	17	82	4050	7.58	NP
6	285	299	238	178	444	950	1620	4014	7.52	NP
7	429	823	401	666	519	859	106	3803	7.12	NP
8	1	35	28	2	0	2023	136	2225	4.17	NP
9	11	1463	260	24	3	118	64	1943	3.64	T&B
10	4	1462	206	17	2	1	2	1694	3.17	Neutrophil
11	0	1496	120	4	11	52	8	1691	3.17	Neutrophil
12	1	5	7	0	0	1372	122	1507	2.82	NP
13	0	1149	43	3	0	0	0	1195	2.24	Neutrophil
14	3	24	28	1	17	1011	53	1137	2.13	Endothelia
15	0	776	188	5	0	0	0	969	1.81	Erythrocyte
16	9	32	15	5	32	535	152	780	1.46	NP
17	1	39	16	1	6	565	40	668	1.25	SMC
18	0	440	189	0	0	0	0	629	1.18	Erythrocyte
19	1	109	49	1	0	123	23	306	0.57	Macrophage
Sum	5340	9339	7120	7206	5376	11346	7669	53396	100.00	
Proposition (%)	10.00	17.49	13.33	13.50	10.07	21.25	14.36	100.00		

Supplementary Table 2: Information of si-RNA target sequence

Species	Si-RNA	Target sequence
Human	Si-SRGN_1	GACCAATGTTCGAACTACT
Human	Si-SRGN_2	CCAGACAGTAATTCTGCAA
Human	Si-SRGN_3	GTCTGAGGACTGACCTTTT
Human	Si-P65_1	GCTGCAGTTTGATGATGAA
Human	Si-P65_1	GCCCTATCCCTTTACGTCA
Human	Si-P65_1	GGACATATGAGACCTTCAA

Supplementary Table 3: Supplementary Antibodies and dilution information

Antibody	Source	Cat# No.	WB	IHC	IF
**Primary antibody**					
UBE2C	Bioss Antibodies	bs-8357R		1:100	
FBLN1	Bioss Antibodies	bs-0809R		1:100	1:100
CHI3L2	Bioss Antibodies	bs-12358R		1:100	
DKK1	Bioss Antibodies	bs-2162R		1:100	
MSMO1	Abcam	ab203587		1:200	
CP	Bioss Antibodies	bs-2373R		1:100	
SRGN	Santa Cruz	HPA000759		1:200	1:100
SRGN	Abcam	ab156991 (not available)	1:1000		
SRGN	Sigma-Aldrich	SAB2103016	1:1000		
COL 1	Bioss Antibodies	bs-10423R		1:100	1:100
COL 2	Cell Signaling Technology	13141			
ACAN	Cell Signaling Technology	3033		1:100	1:100
IL-1β	Abcam	ab254360	1:1000		1:50
IL-1β	Abcam	ab283818		1:200	
CCL3	Abcam	ab259372	1:1000	1:200	1:500
TNF-α	Abcam	ab183218	1:1000		
TNF-α	Abcam	ab1793		1:200	1:100
IKBα	Abcam	ab32518	1:1000		
p- IKBα	Abcam	ab133462	1: 1000		
pan-AKT	Abcam	ab8805	1: 1000		
p-AKT	Abcam	ab8933	1: 1000		
Smad2	Abcam	ab40855	1: 1000		
p-Smad2	Abcam	ab280888	1: 1000		

Smad3	Abcam	ab40854	1: 1000		
p-Smad3	Abcam	ab52903	1: 1000		
ERK1/2	Abcam	ab184699	1: 1000		
p-ERK1/2	Abcam	ab201015	1: 1000		
p65	Abcam	ab16502	1:1000		
p-p65	Santa Cruz	sc-136548	1:1000		1:100
p50/p105	Abcam	ab305263	1: 1000		
p52/ p100	Santa Cruz	sc-7386	1: 1000		
cRel	Abcam	ab133251	1: 1000		
RelB	Abcam	ab33907	1:1000		
F4/80	Bioss Antibodies	bsm-34028M		1:100	1:100
CD86	Bioss Antibodies	bs-1035R			1:100
Beta Tubulin (HRP conjugated)	Bioss Antibodies	bsm-52847R	1:5000		

**Flow Cytometry Antibody**					
CD11c-PC7	eBioScience	25-0114-81			
CD86-PB450	eBioScience	48-0862-80			

**Secondary antibody**					
Anti-rabbit IgG, HRP-linked Antibody	Cell Signaling Technology	7074S	1:5000		
Anti-mouse IgG, HRP-linked Antibody	Cell Signaling Technology	7076S	1:5000		

Goat anti-Rabbit IgG (H+L) Cross-Adsorbed Secondary Antibody, Alexa Fluor™ 488	Thermo Fisher Scientific	A-11008	1:2000
Goat anti-mouse IgG (H+L) Secondary Antibody, DyLight™ 488	Thermo Fisher Scientific	A-10680	1:2000
Goat anti-Rabbit IgG (H+L) Highly Cross-Adsorbed Secondary Antibody, Alexa Fluor™ 555	Thermo Fisher Scientific	A-21429	1:2000
Goat anti-Rabbit IgG (H+L) Cross-Adsorbed Secondary Antibody, Alexa Fluor™ 594	Thermo Fisher Scientific	R-37117	1:2000
Goat anti-Rabbit IgG (H+L) Highly Cross-Adsorbed Secondary Antibody, Alexa Fluor™ 647	Thermo Fisher Scientific	A-21245	1:2000

Supplementary Table 4: Main Reagents information

Reagents	Source	Cat# No.	Application
PrimeScript™ II Reverse Transcriptase	TaKaRa	2690A	RT-qPCR
TB Green® Premix Ex Taq™ II (Tli RNaseH Plus)	TaKaRa	RR820A	RT-qPCR
DAKO REALTM EnVision^{TM} Detection system	DAKO	K5007	IHC
Lipofectamine™ 3000 Transfection Reagent	Invitrogen, Thermo Fisher Scientific	L3000015	Si-RNA, plasmid transfection
Recombined human SRGN protein	R&D	10190-SN-050	
Daphetin	Abcam	ab143113	
Human IL-1 beta/IL-1F2 Quantikine ELISA Kit	R&D Systems	MLB00C	ELISA
Human TNF-alpha Quantikine ELISA Kit	R&D Systems	DTA00D	ELISA
Human CCL3/MIP-1 alpha Quantikine ELISA Kit	R&D Systems	DMA00	ELISA

Supplementary Table 5: Cell line authentication report of RAW264.7

The STR typing results of RAW264.7 cells and their matching information in the cell repository*

Locus	Cell Sample**		RAW264.7 in Cellosaurus release 43.0
**4-2**	246.91 [22.3]		223
**5-5**	335.85 [14]		14
**6-4**	299.98 [18]		18
**6-7**	336.28 [12]		12
**9-2**	222.32 [15]		15
**12-1**	225.93 [16]		16
**15-3**	201 [22.3]	205.05 [23.3]	22.3
**18-3**	161.17 [18]		18
**X-1**	396.95 [24]		24
**D4S2408**	/		/

*Cell line STR authentication report No. 20210203shu-2; Based on the cell DNA typing results, the identification of this cell strain is confirmed to be of murine origin. The DNA typing showed a close match (EV value 0.9412) with a cell line found in the cell repository, identified as ImKC/RAW264.7, with the accession number CVCL HF55/CVCL 0493. No multiple allele phenomena were observed, and there was no evidence of human cross-contamination. (The D4S2408 locus is a human-specific marker used to detect potential human contamination in the cell line.)

**Sample name: RAW264.7; Date: Feb. 3,2021 ; Sample treatment: DNA extraction was performed from a cell pellet containing 1× 106 cells using Axygen's genome extraction kit. The extracted DNA was then subjected to amplification following the 10-STR amplification protocol,

and the STR loci, along with the gender gene Amelogenin, were subsequently analyzed using the ABI 3730XL genetic analyzer.

Supplementary figures

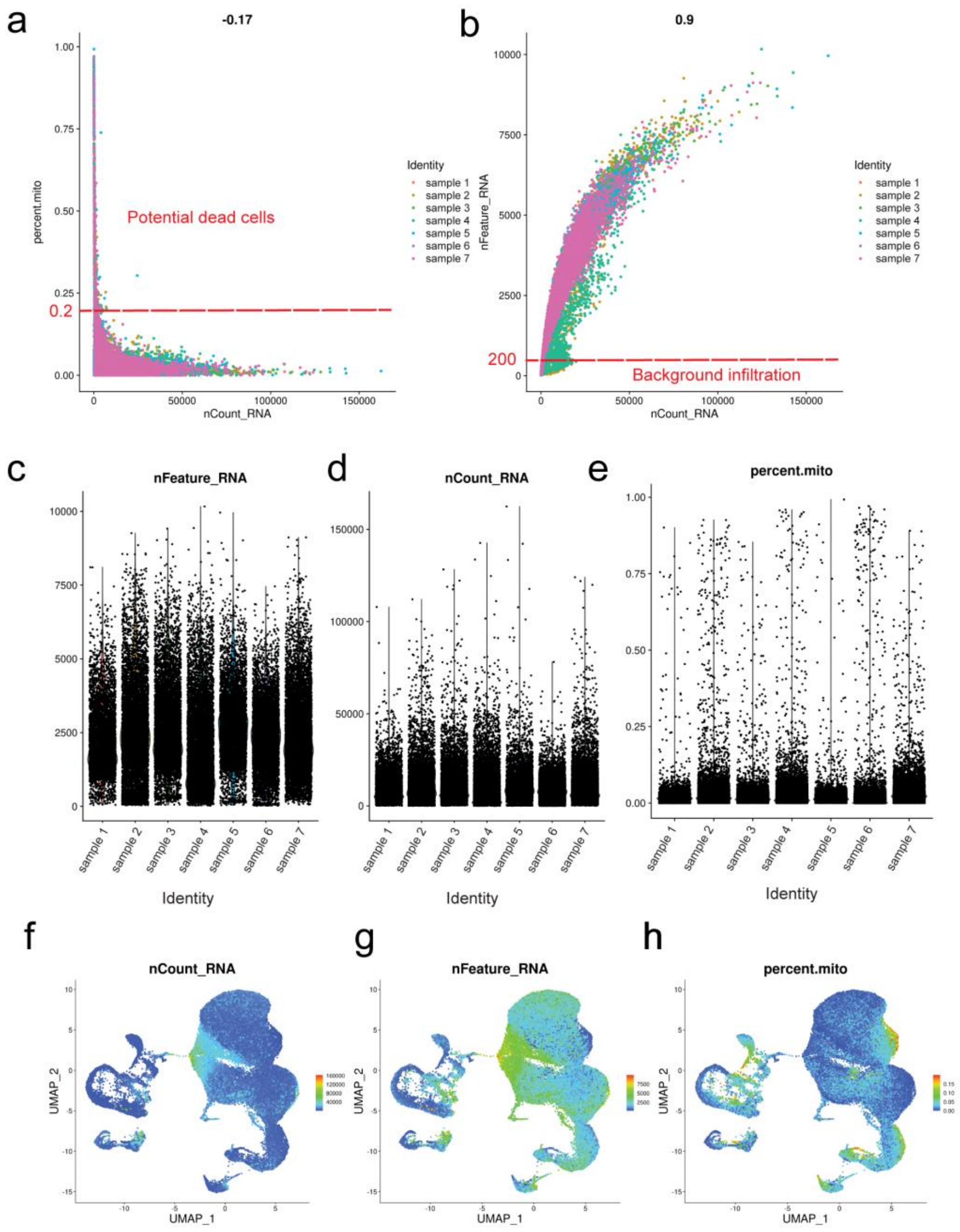

Supplementary Figure 1. Single-cell sequencing of cell infiltration. By visualizing the gene count distribution plots and unique molecular identifier (UMI) count distribution plots, the expression profile and gene abundance in all cells in the sample can be assessed.

(a and b) Correlation analysis of UMI with genes and mitochondrial proportion. **(c-h)** Violin and UMAP plots of all single-cell transcriptomes colored by individual participants.

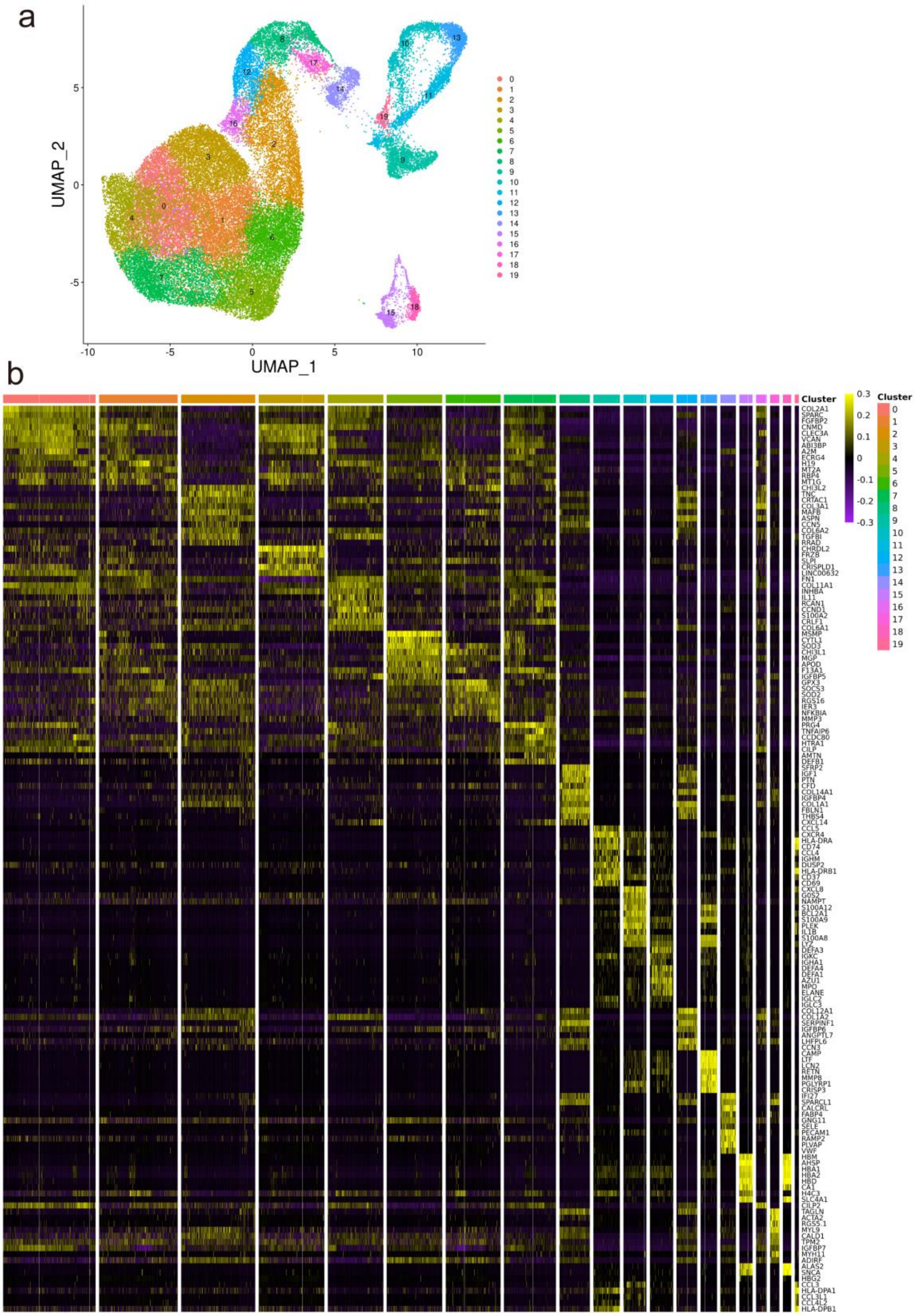
a
b
UMAP_1
UMAP_2
Cluster

Supplementary Figure 2. Cell clustering analysis (a) Umap of 20 cell populations in the cell clustering analysis. **(B)** Heatmap revealing the scaled expression of differentially expressed genes for each cell population.

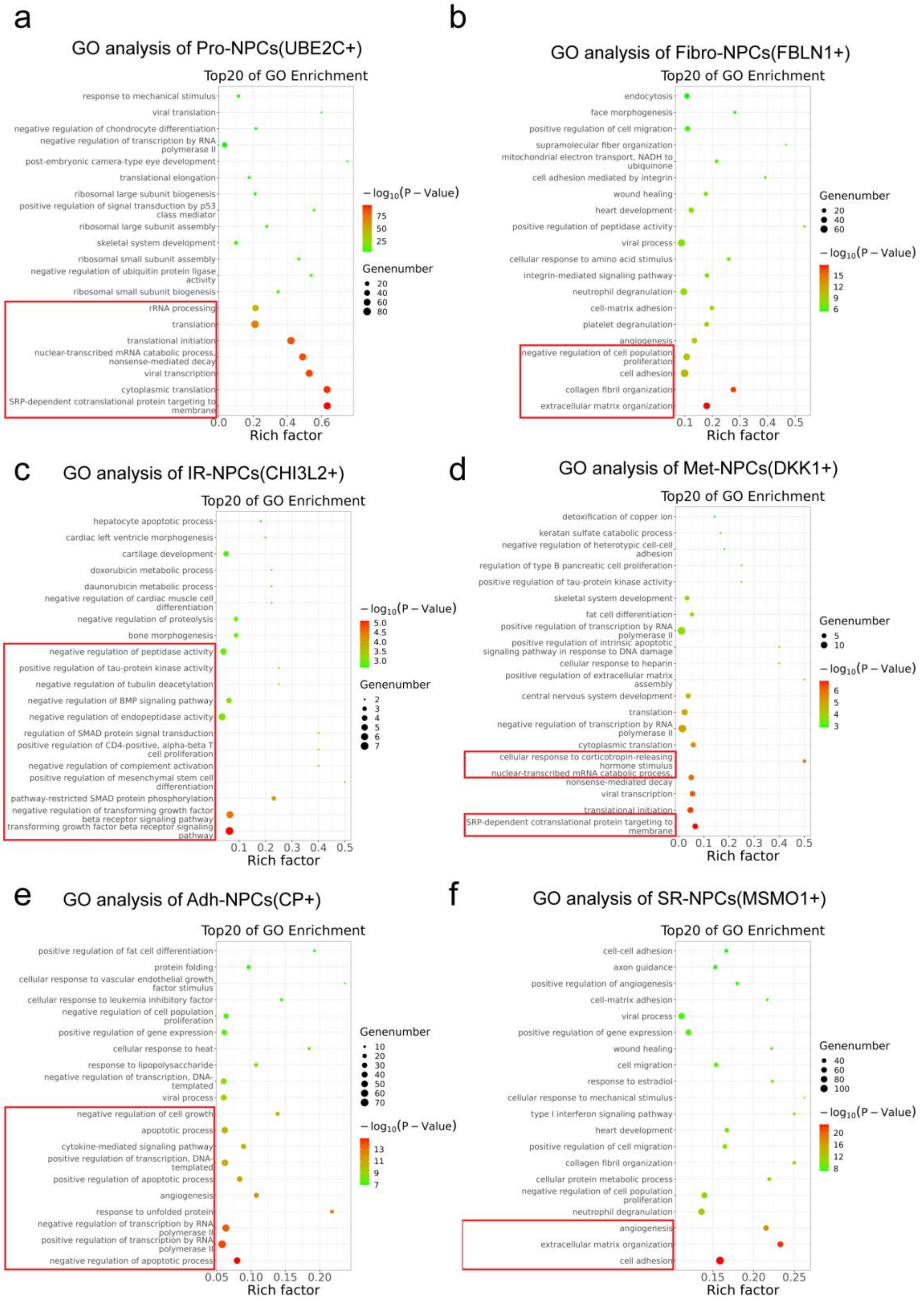
a
GO analysis of Pro-NPCs(UBE2C+)
Top20 of GO Enrichment
response to mechanical stimulus
viral translation
negative regulation of chondrocyte differentiation
negative regulation of transcription by RNA polymerase II
post-embryonic camera-type eye development
translational elongation
ribosomal large subunit biogenesis
positive regulation of signal transduction by p53 class mediator
ribosomal large subunit assembly
skeletal system development
ribosomal small subunit assembly
negative regulation of ubiquitin protein ligase activity
ribosomal small subunit biogenesis
rRNA processing
translation
translational initiation
nuclear-transcribed mRNA catabolic process, nonsense-mediated decay
viral transcription
cytoplasmic translation
SRP-dependent cotranslational protein targeting to membrane
Rich factor
b
GO analysis of Fibro-NPCs(FBLN1+)
Top20 of GO Enrichment
endocytosis
face morphogenesis
positive regulation of cell migration
supramolecular fiber organization
mitochondrial electron transport, NADH to ubiquinone
cell adhesion mediated by integrin
wound healing
heart development
positive regulation of peptidase activity
viral process
cellular response to amino acid stimulus
integrin-mediated signaling pathway
neutrophil degranulation
cell-matrix adhesion
platelet degranulation
angiogenesis
negative regulation of cell population proliferation
cell adhesion
collagen fibril organization
extracellular matrix organization
Rich factor
c
GO analysis of IR-NPCs(CHI3L2+)
Top20 of GO Enrichment
hepatocyte apoptotic process
cardiac left ventricle morphogenesis
cartilage development
doxorubicin metabolic process
daunorubicin metabolic process
negative regulation of cardiac muscle cell differentiation
negative regulation of proteolysis
bone morphogenesis
negative regulation of peptidase activity
positive regulation of tau-protein kinase activity
negative regulation of tubulin deacetylation
negative regulation of BMP signaling pathway
negative regulation of endopeptidase activity
regulation of SMAD protein signal transduction
positive regulation of CD4-positive, alpha-beta T cell proliferation
negative regulation of complement activation
positive regulation of mesenchymal stem cell differentiation
pathway-restricted SMAD protein phosphorylation
negative regulation of transforming growth factor beta receptor signaling pathway
transforming growth factor beta receptor signaling pathway
Rich factor
d
GO analysis of Met-NPCs(DKK1+)
Top20 of GO Enrichment
detoxification of copper ion
keratan sulfate catabolic process
negative regulation of heterotypic cell-cell adhesion
regulation of type B pancreatic cell proliferation
positive regulation of tau-protein kinase activity
skeletal system development
fat cell differentiation
positive regulation of transcription by RNA polymerase II
positive regulation of intrinsic apoptotic signaling pathway in response to DNA damage
cellular response to heparin
positive regulation of extracellular matrix assembly
central nervous system development
translation
negative regulation of transcription by RNA polymerase II
cytoplasmic translation
cellular response to corticotropin-releasing hormone stimulus
nuclear-transcribed mRNA catabolic process, nonsense-mediated decay
viral transcription
translational initiation
SRP-dependent cotranslational protein targeting to membrane
Rich factor
e
GO analysis of Adh-NPCs(CP+)
Top20 of GO Enrichment
positive regulation of fat cell differentiation
protein folding
cellular response to vascular endothelial growth factor stimulus
cellular response to leukemia inhibitory factor
negative regulation of cell population proliferation
positive regulation of gene expression
cellular response to heat
response to lipopolysaccharide
negative regulation of transcription, DNA-templated
viral process
negative regulation of cell growth
apoptotic process
cytokine-mediated signaling pathway
positive regulation of transcription, DNA-templated
positive regulation of apoptotic process
angiogenesis
response to unfolded protein
negative regulation of transcription by RNA polymerase II
positive regulation of transcription by RNA polymerase II
negative regulation of apoptotic process
Rich factor
f
GO analysis of SR-NPCs(MSMO1+)
Top20 of GO Enrichment
cell-cell adhesion
axon guidance
positive regulation of angiogenesis
cell-matrix adhesion
viral process
positive regulation of gene expression
wound healing
cell migration
response to estradiol
cellular response to mechanical stimulus
type I interferon signaling pathway
heart development
positive regulation of cell migration
collagen fibril organization
cellular protein metabolic process
negative regulation of cell population proliferation
neutrophil degranulation
angiogenesis
extracellular matrix organization
cell adhesion
Rich factor
Genenumber
−log10(P−Value)

Supplementary Figure 3. GO analysis of the six subpopulations of NPCs (a-f) GO analysis of the six subpopulations of NPCs in scRNA-seq results.

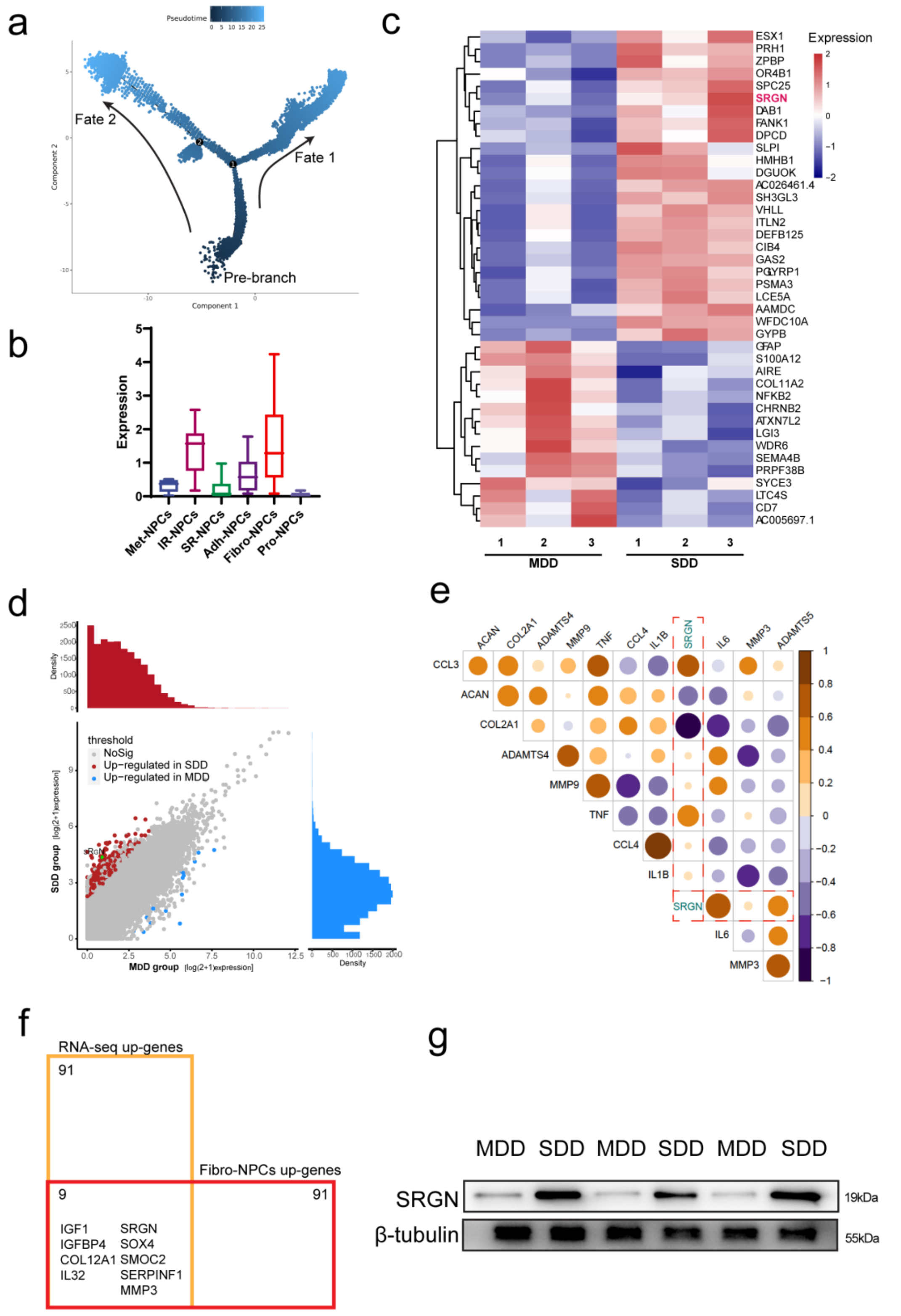

Supplementary Figure 4. SRGN is a potential biomarker of late-stage NPCs (a) Monocle 2 pseudotime trajectory analysis and state distribution. **(b)** Box plot of SRGN gene expression in each subcluster. **(c and d)** Heatmap and volcano plot of 6

human NP samples (mild degenerative disc [MDD]: n = 3, severe degenerative disc [SDD]: n = 3). **(e)** Correlation analysis of SRGN and IVDD-related markers. **(f)** Venn diagram of the intersection of the top 100 upregulated genes in RNA-Seq of human NP tissues and the top 100 significant marker genes in Fibro-NPCs. **(g)** Western blotting analysis of SRGN expression in normal NP compared to degenerated NP samples. M: MDD group; S: SDD group.

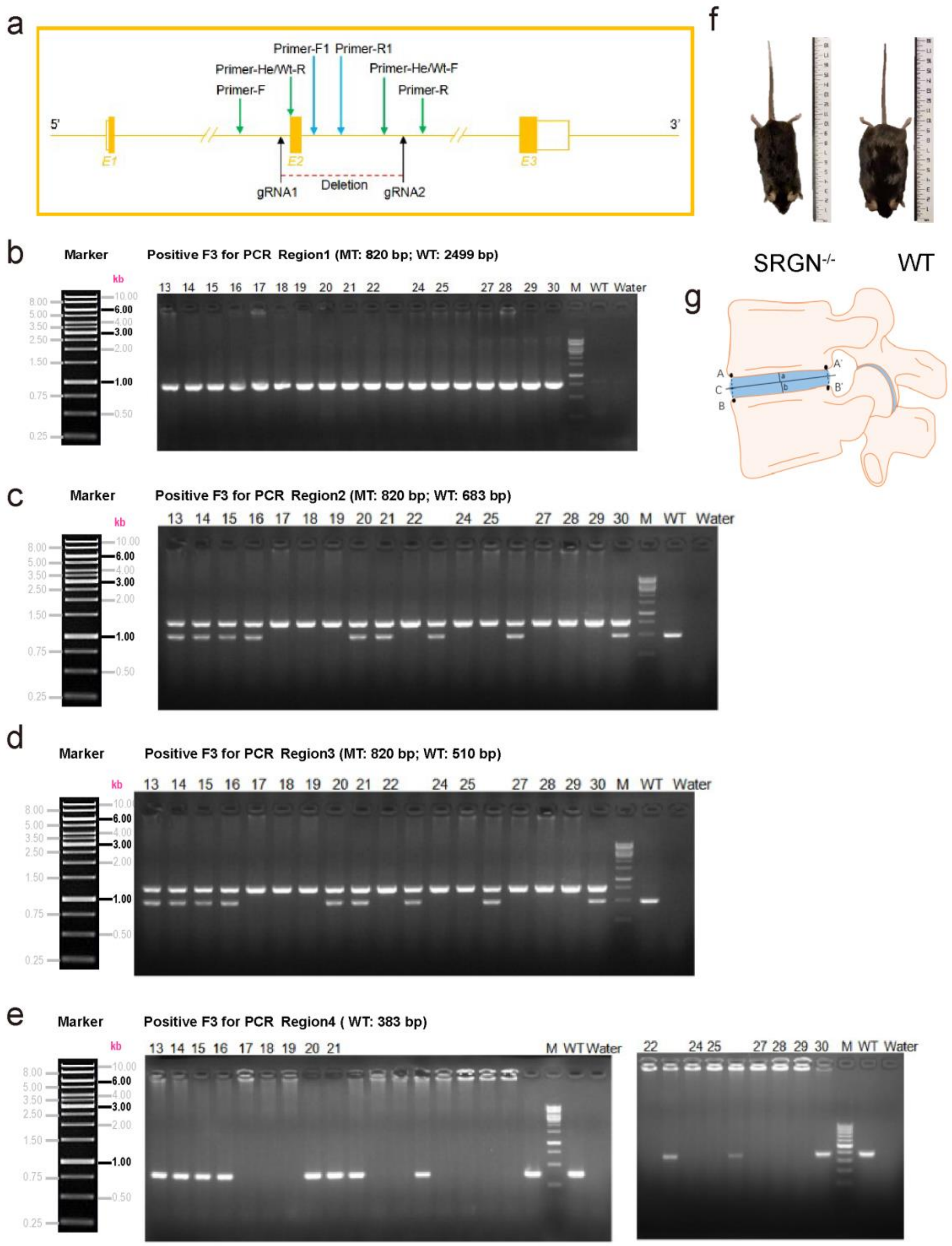

Supplementary Figure 5. Successful creation of SRGN KO mice (a) Schematic diagram illustrating the targeting strategy. **(b- e)** PCR screening results of F3 animals from 4 different regions to identify KO mice and WT mice. (PCR primer sequences are shown at the end of the document. MT: mutant allele.) **(f)** The appearance of male KO mice and WT mice. **(g)** The method of intervertebral disk height measurement was as follows: Determine the four corners of the two adjacent

vertebral bodies (A, A ', B, B ') and draw a straight line through the midpoints of A and B and the midpoints of A 'and B', namely, the bisector (line C). The vertical distance between A, A' and B and the midpoint of B' to line C is A and B, respectively, and the sum of the two (A + B) is the disc height.

Sequence of gRNA and PCR primers

1.gRNA target sequence
gRNA1 (matches reverse strand of gene): TTGTACATTTGGCAGGTCGCAGG
gRNA2 (matches forward strand of gene): CACGGGAATAAGTTGTTGCCTGG
2.PCR screening
PCR primers 1 (annealing temperature 65.0 ºC):
Forward primer-F: GTGTCTGTCACAGGCATTGTTGG
Reverse primer-R: TACTGATGGGAGATGCCAGACCC
Mutant allele: 820 bp
Wildtype allele: 2499 bp
PCR region 2 (annealing temperature 60.0 ºC):
Mouse Srgn-F: GTGTCTGTCACAGGCATTGTTGG
Mouse Srgn-R: TACTGATGGGAGATGCCAGACCC
Mouse Srgn-He/Wt-R: GATAACCTGTGCGAACAAGGAAC
Heterozygote: 820 bp and 683 bp;
Homozygous: 820 bp
Wildtype: 683 bp
PCR region 3 (annealing temperature 60.0 ºC):
Mouse Srgn-F: GTGTCTGTCACAGGCATTGTTGG
Mouse Srgn-R: TACTGATGGGAGATGCCAGACCC
Mouse Srgn-He/Wt-F: ACCACCACACAATGATATACAAACAGAT
Heterozygous: 820 bp and 510 bp;
Homozygous: 820 bp
Wildtype: 510 bp
PCR region 4 (annealing temperature 60.0 ºC):
Mouse Srgn-F1: ATAAGCCTTGGAAAGTATTTGCCC
Mouse Srgn-R1: GAAGCAAAGTCAAAGGGTCCATTT
Heterozygous: 383 bp;
Homozygous: 0 bp
Wildtype: 383 bp

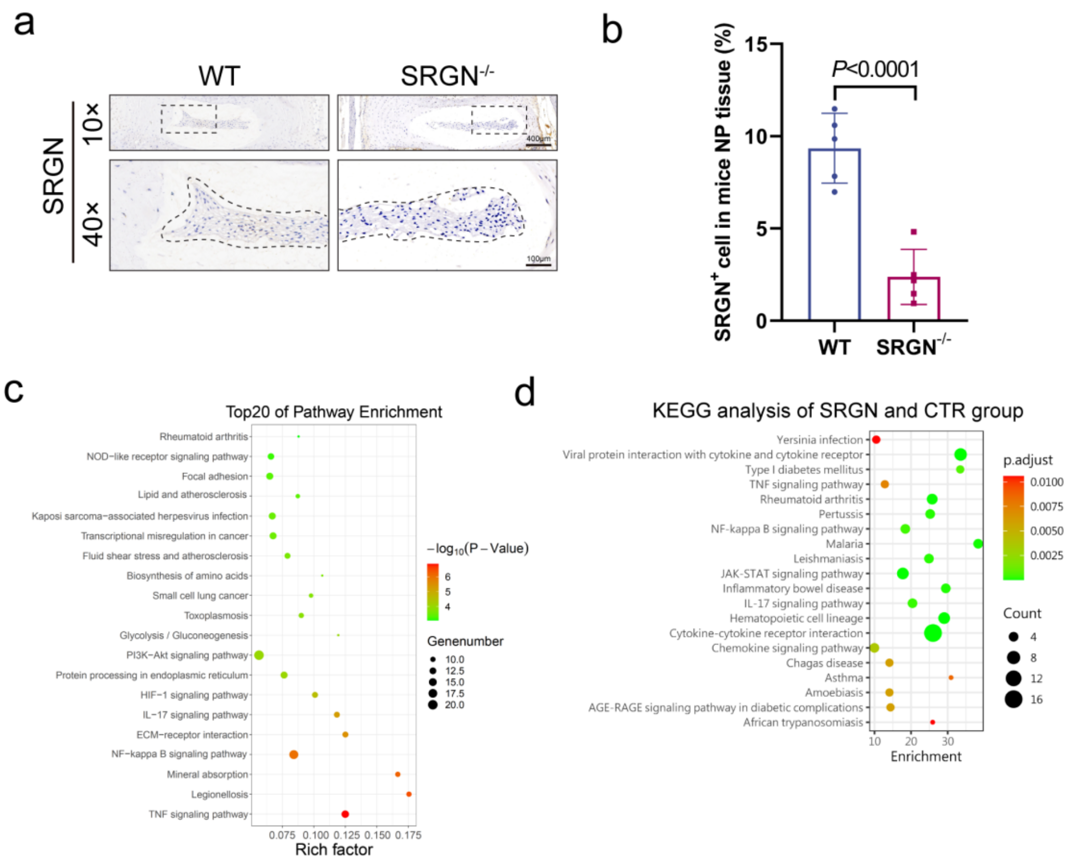

Supplementary Figure 6. SRGN regulates the local inflammatory response through the NF-κB signaling pathway (a and b) The IHC staining and analysis of WT and *Srgn*$^{-/-}$ mouse IVDs. (c)KEGG analysis of the top 20 upregulated genes in SDD based on RNA-seq. **(d)** KEGG analysis of SRGN-treated NPC inflammatory cytokines from the cytokine array. Data are presented as mean ± SD. Statistical significance was determined by two-tailed t test. Source data are provided as a Source Data file.

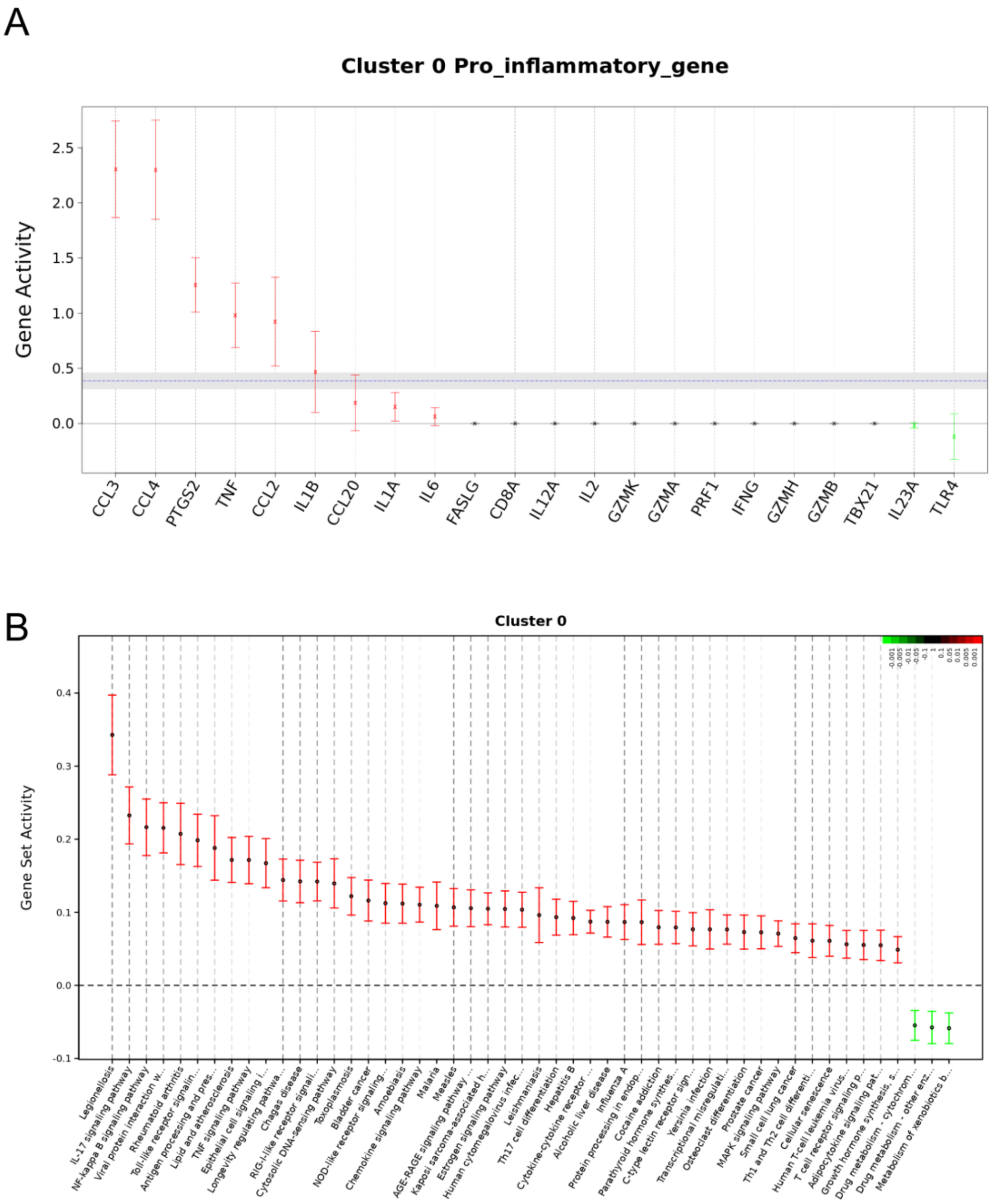

Supplementary Figure 7. SRGN increases macrophage infiltration by activating the NF-κB signaling pathway. **(a)** Proinflammatory gene set expression in cluster 0 indicates that CCL3, IL-1β and TNF-α gene expression is increased significantly. **(b)** QuSage analysis showed that the NF-κB signaling pathway is significant in cluster 0 macrophages.

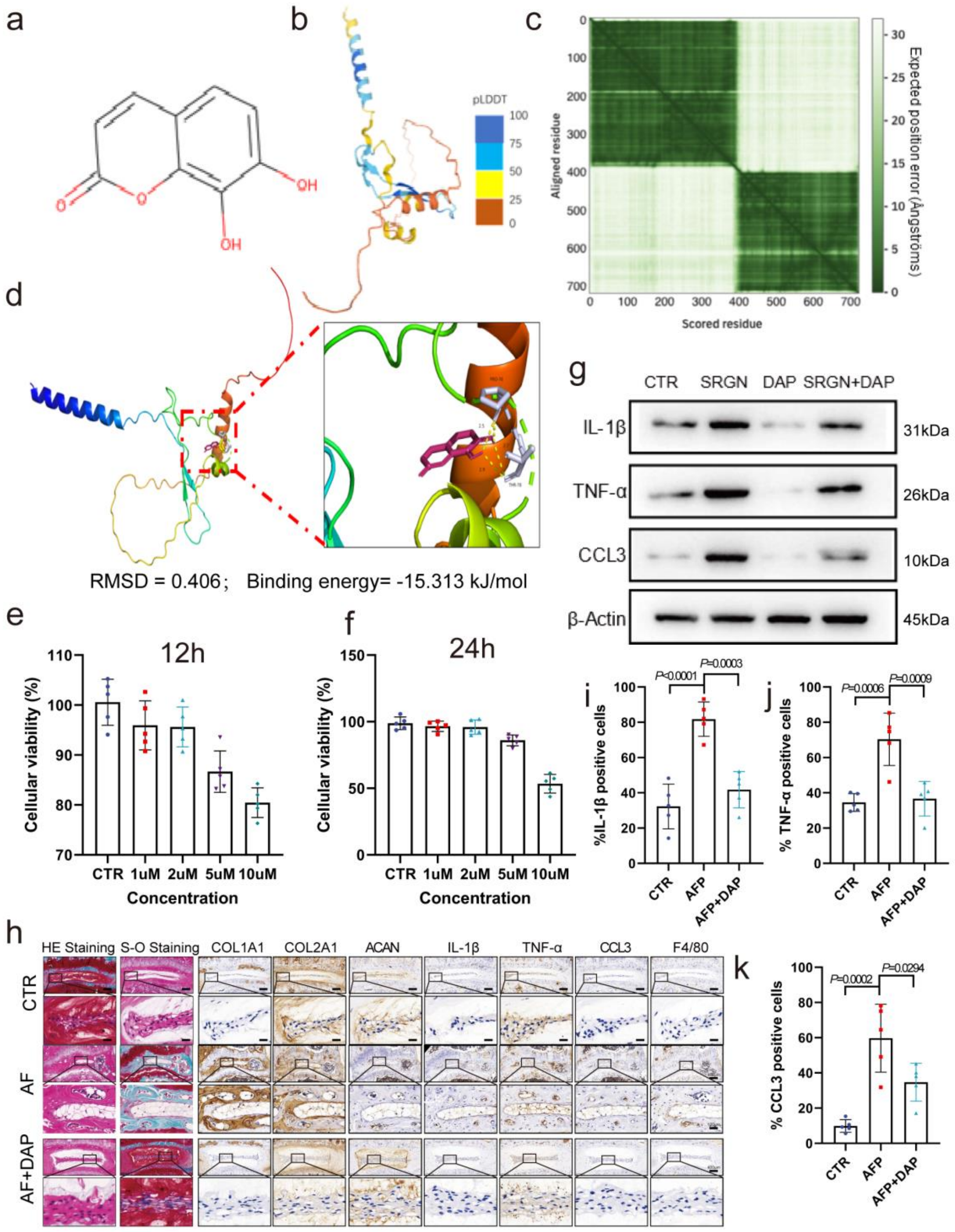

Supplementary Figure 8. DAP attenuates the IVD local inflammatory response to alleviate IVDD.

(a) The structure of DAP. **(b)** The predicted 3D structure of SRGN by AlphaFold 2.

(c) Predicted aligned error of the SRGN 3D model: The color at position (x, y) indicates AlphaFold’s expected position error at residue x when the predicted and true structures are aligned on residue y. **(d)** Simulation of predicted docking with SRGN

and DAP (RMSD = 0.406; estimated free energy of binding = -15.313 kJ/mol). **(e and f)** CCK-8 assay of DAP and 2 μM with 24 h is the best treated concentration and time. **(g)** Western blotting analysis of IL-1β, TNF-α, and CCL3 in NPCs treated with SRGN and DAP. **(h)** HE, SO and IHC staining of COL1A1, COL2A1, ACAN, IL-1β, TNF-α, CCL3, and F4/80 with histologic score analysis of IL-1β+, TNF-α+, CCL3+, and F4/80+ cells in AF and AF plus DAP mice at 8 weeks after the operation (original magnification 100×, 400×, scale bar = 400 μm, 100 μm). Data are represented as mean ± standard deviation. *P* values were determined by one-way ANOVA with post-hoc Bonferroni correction or Kruskal-Wallis H test with a Dunn's correction as appropriate.

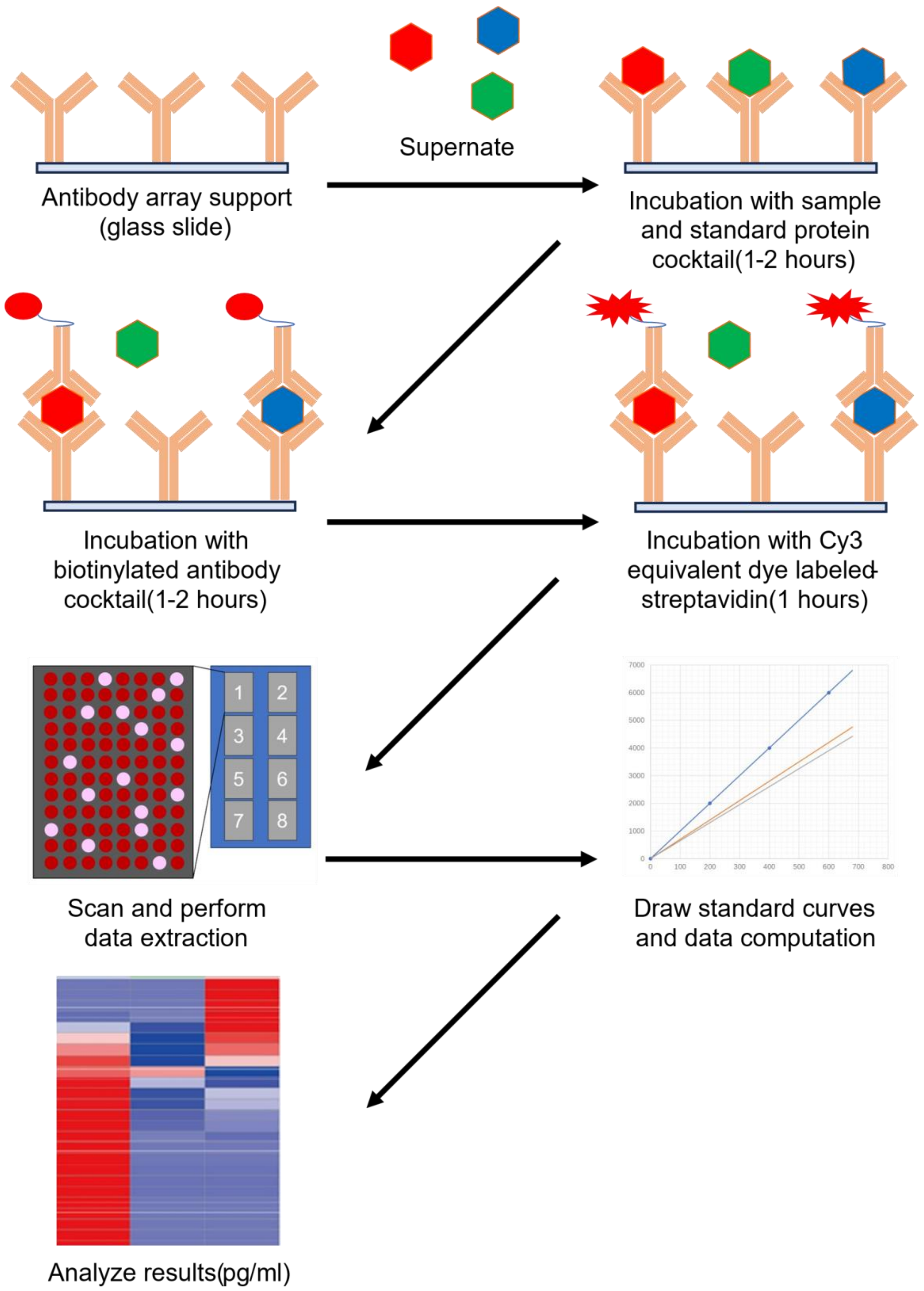

Supplementary Figure 9. Schematic diagram of the quantitative measurement of 40 human cytokines.

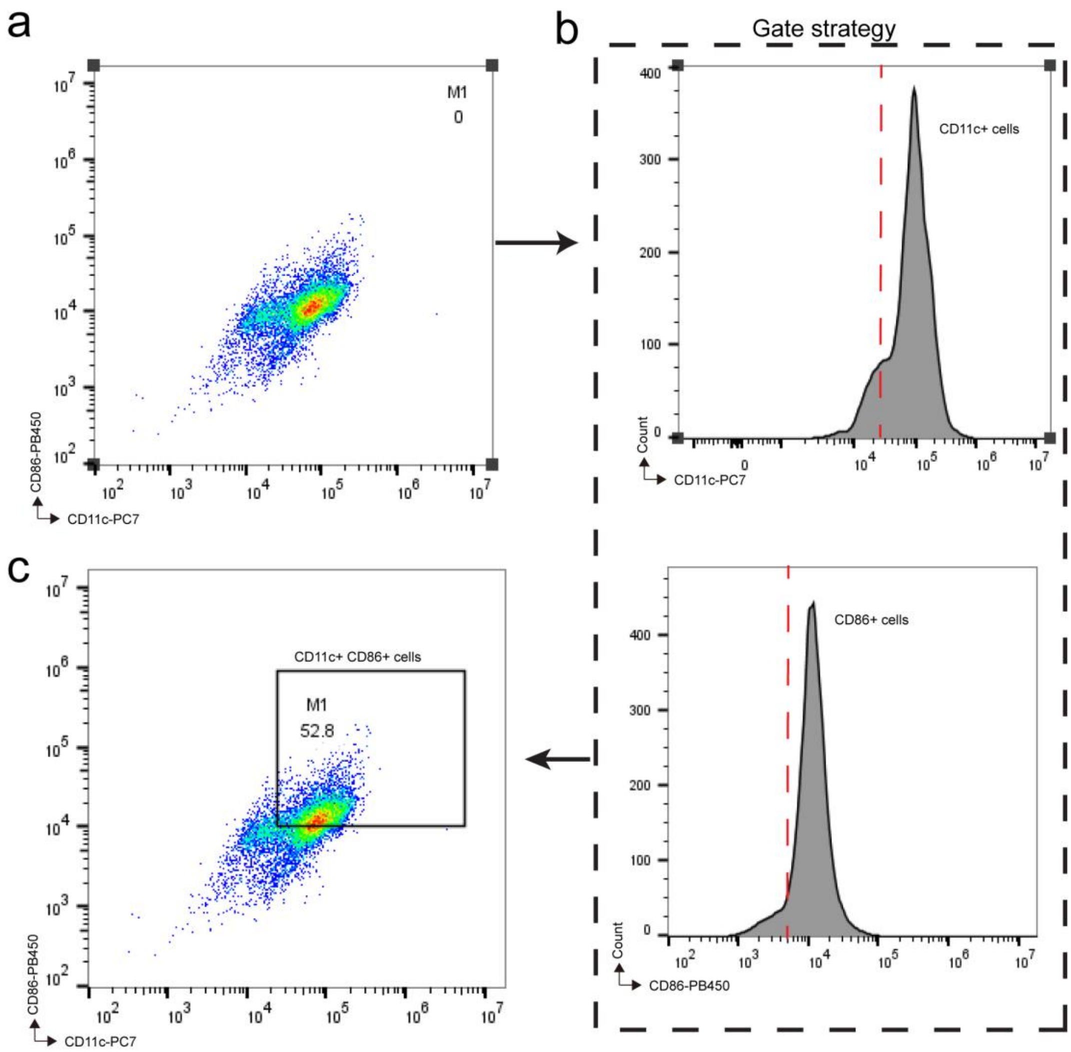

Supplementary Figure 10. Gating strategy for screening CD11c and CD86 positive macrophages.
