# Supplementary material for: Serglycin secreted by late-stage nucleus pulposus cells is a biomarker of intervertebral disc degeneration
Source: Nat Commun. 2024 Jan 2;15:47. doi: 10.1038/s41467-023-44313-9 (PMC10761730; doi:10.1038/s41467-023-44313-9)
Supplement: Supplementary file 2 — Reporting Summary [file 41467_2023_44313_MOESM2_ESM.pdf]

nature portfolio

Corresponding author(s): Zhaomin Zheng and Jianru Wang

Last updated by author(s): Oct 11, 2023

Reporting Summary

Nature Portfolio wishes to improve the reproducibility of the work that we publish. This form provides structure for consistency and transparency in reporting. For further information on Nature Portfolio policies, see our Editorial Policies and the Editorial Policy Checklist.

Statistics

For all statistical analyses, confirm that the following items are present in the figure legend, table legend, main text, or Methods section.

n/a	Confirmed	
☐	☒	The exact sample size (*n*) for each experimental group/condition, given as a discrete number and unit of measurement
☐	☒	A statement on whether measurements were taken from distinct samples or whether the same sample was measured repeatedly
☐	☒	The statistical test(s) used AND whether they are one- or two-sided<br>*Only common tests should be described solely by name; describe more complex techniques in the Methods section.*
☒	☐	A description of all covariates tested
☐	☒	A description of any assumptions or corrections, such as tests of normality and adjustment for multiple comparisons
☐	☒	A full description of the statistical parameters including central tendency (e.g. means) or other basic estimates (e.g. regression coefficient) AND variation (e.g. standard deviation) or associated estimates of uncertainty (e.g. confidence intervals)
☒	☐	For null hypothesis testing, the test statistic (e.g. *F*, *t*, *r*) with confidence intervals, effect sizes, degrees of freedom and *P* value noted<br>*Give P values as exact values whenever suitable.*
☒	☐	For Bayesian analysis, information on the choice of priors and Markov chain Monte Carlo settings
☒	☐	For hierarchical and complex designs, identification of the appropriate level for tests and full reporting of outcomes
☒	☐	Estimates of effect sizes (e.g. Cohen's *d*, Pearson's *r*), indicating how they were calculated

Our web collection on statistics for biologists contains articles on many of the points above.

Software and code

Policy information about availability of computer code

Data collection

1. micro CT image collection: SkyScan 1176 (Bruker, Kartuizersweg, Belgium)
2. microscopy images of HE, SO and IHC staining: Kfbio/KF-PRO-020 Digital pathological scanner (Zhejiang, China)
2. microscopy images of tissue Immunofluorescence:Olympus BX63 Microscopes(
3. microscopy images of cell Immunofluorescence analysis: confocal microscope(Lecia, Germany)
3.RT-qPCR data collection: Applied Biosystems 7500 Sequencing Detection System (version. 2.3; Foster City, CA, USA)
4. Flow Cytometer: Beckman CytExpert analyzer.
5. Western blot images: Tanon 4600 (Shanghai, China)

Data analysis

We used GraphPad Prism (version 8.0) for statistical analysis;
FlowJo_v10.8.1 for flow cytometry analysis;
ImageJ 1.50i for microscopy images analysis;
SkyScan1276_CTAn for disc height analysis;
Alphafold 2 for Serglycin protein structure predicted;
AutodockTools_1.5.6 and Pymol v1.8 for molecular docking.

scRNA-seq data analysis was performed by NovelBio Bio-Pharm Technology Co.,Ltd. with NovelBrain Cloud Analysis Platform.
Cytokine Array analysis was performed by The RayBio® Analysis Tool (RayBio®, Norcross, GA)
RNA-seq analysis was performed by Guangzhou Epibiotek Co., Ltd., Guangzhou, China
the package are follows:

HISAT2 (version 2 2.1.0): https://daehwankimlab.github.io/hisat2/download/#version-hisat2-210
Htseq (Version 0.11.0): https://htseq.readthedocs.io/en/release_0.11.1/install.html
DESeq2 (1.22.2):https://bioconductor.org/packages/release/bioc/html/DESeq2.html
ggplot2: https://cran.r-project.org/web/packages/ggplot2/index.html
sankey plot: https://r-charts.com/flow/sankey-diagram-ggplot2/
Venn plot: ggsankey packages: https://r-charts.com/part-whole/ggvenndiagram/
Circos Plot: ggVennDiagram packages : https://cran.r-project.org/web/packages/RCircos/
corrplot: corrplot packages: https://cran.r-project.org/web/packages/corrplot/index.html

For manuscripts utilizing custom algorithms or software that are central to the research but not yet described in published literature, software must be made available to editors and reviewers. We strongly encourage code deposition in a community repository (e.g. GitHub). See the Nature Portfolio guidelines for submitting code & software for further information.

Data

Policy information about availability of data

All manuscripts must include a data availability statement. This statement should provide the following information, where applicable:

- Accession codes, unique identifiers, or web links for publicly available datasets
- A description of any restrictions on data availability
- For clinical datasets or third party data, please ensure that the statement adheres to our policy

The authors declare that the data supporting the findings of this study are available with the paper and its Supplementary information files. All data are available in the main text or the materials. The raw sequence data reported in this paper have been deposited in the GEO database under accession code GSE244889(https://www.ncbi.nlm.nih.gov/geo/query/acc.cgi?acc=GSE244889). The data supporting the findings of this study are available from the corresponding authors upon reasonable request. Source data are provided with this paper.

Human research participants

Policy information about studies involving human research participants and Sex and Gender in Research.

Reporting on sex and gender	No sex- and gender-based analyses have been performed.<br>there was no significant statistical significance between the male and female for the incidence rate of intervertebral disc degeneration
Population characteristics	Human NP tissue samples were collected from patients (19 females and 29 males, age 10-85 years old) undergoing NP removal. The degree of disc degeneration was evaluated according to the Pfirrmann grading system. Normal IVDs were obtained from patients who had experienced trauma, and degenerated IVDs were obtained from patients with degenerative spinal diseases (disc herniation, spinal canal stenosis, and degenerative scoliosis).
Recruitment	All the patients signed an informed consent form. NP samples from Pfirrmann grade I-II were included into the mild degenerated discs (MDD) group; samples from Pfirrmann grade III-V were included into the severe degenerative discs (SDD) group. The information of human NP samples were listed in the supplementary tables. Between September 2016 and June 2021, we collected 48 IVD samples from patients (19 females and 29 males, age 10-85 years old). Detailed information on the specimens is shown in Table S2.
Ethics oversight	This study protocol of using patient samples was approved by the Ethics Committee of The First Affiliated Hospital of Sun Yat-sen University.

Note that full information on the approval of the study protocol must also be provided in the manuscript.

Field-specific reporting

Please select the one below that is the best fit for your research. If you are not sure, read the appropriate sections before making your selection.

☒ Life sciences ☐ Behavioural & social sciences ☐ Ecological, evolutionary & environmental sciences

For a reference copy of the document with all sections, see nature.com/documents/nr-reporting-summary-flat.pdf

Life sciences study design

All studies must disclose on these points even when the disclosure is negative.

Sample size	Sample size was chosen to ensure an adequate statistical power. For in vitro study, 5 biological replicates were used. The number of animal were described in the manuscript.
Data exclusions	Samples lacking Intervertebral disc tissue in the spine slide were excluded for the tissue analysis
Replication	All experimental findings were reproduced in dependently as least three times. The number of biologically independent samples, mice per

	group, or human specimens were listed in the figure legend. Quantitative data shown as mean ± s.d.
Randomization	Human or mouse NP tissue were randomly assigned to each experimental group. Animals were also randomly assigned to each experimental group
Blinding	Pfirrmann grade, H&E score evaluation, disc height evaluation were performed by individuals who were blinded to the specific conditions of the experimental group.

Reporting for specific materials, systems and methods

We require information from authors about some types of materials, experimental systems and methods used in many studies. Here, indicate whether each material, system or method listed is relevant to your study. If you are not sure if a list item applies to your research, read the appropriate section before selecting a response.

Materials & experimental systems

n/a	Involved in the study	
☐	☒	Antibodies
☐	☒	Eukaryotic cell lines
☒	☐	Palaeontology and archaeology
☐	☒	Animals and other organisms
☒	☐	Clinical data
☒	☐	Dual use research of concern

Methods

n/a	Involved in the study	
☒	☐	ChIP-seq
☐	☒	Flow cytometry
☒	☐	MRI-based neuroimaging

Antibodies

Antibodies used

All antibodies used in this study are detailed in material and methods section.
The detailed information:(also listed in supplementary tables)
Primary antibody
UBE2C Bioss Antibodies bs-8357R IHC 1:100
FBLN1 Bioss Antibodies bs-0809R IHC 1:100 IF 1:100
CHI3L2 Bioss Antibodies bs-12358R IHC 1:100
DKK1 Bioss Antibodies bs-2162R IHC 1:100
MSMO1 Abcam ab203587 IHC 1:200
CP Bioss Antibodies bs-2373R IHC 1:100
SRGN Santa Cruz HPA000759 IHC 1:200 IF 1:100
SRGN Abcam ab156991
(not available) WB 1:1000
SRGN Sigma-Aldrich SAB2103016 WB 1:1000
COL 1 Bioss Antibodies bs-10423R IHC 1:100 IF 1:100
COL 2 Cell Signaling Technology 13141
ACAN Cell Signaling Technology 3033 IHC 1:100 IF 1:100
IL-1β Abcam ab254360 WB 1:1000 IF 1:50
IL-1β Abcam ab283818 IHC 1:200
CCL3 Abcam ab259372 WB 1:1000 IHC 1:200 IF 1:500
TNF-α Abcam ab183218 WB 1:1000
TNF-α Abcam ab1793 IHC 1:200 IF 1:100
IKBα Abcam ab32518 WB 1:1000
p- IKBα Abcam ab133462 WB 1: 1000
pan-AKT Abcam ab8805 WB 1: 1000
p-AKT Abcam ab8933 WB 1: 1000
Smad2 Abcam ab40855 WB 1: 1000
p-Smad2 Abcam ab280888 WB 1: 1000
Smad3 Abcam ab40854 WB 1: 1000
p-Smad3 Abcam ab52903 WB 1: 1000
ERK1/2 Abcam ab184699 WB 1: 1000
p-ERK1/2 Abcam ab201015 WB 1: 1000
p65 Abcam ab16502 WB 1:1000
p-p65 Santa Cruz sc-136548 WB 1:1000 IF 1:100
p50/p105 Abcam ab305263 WB 1: 1000
p52/ p100 Santa Cruz sc-7386 WB 1: 1000
cRel Abcam ab133251 WB 1: 1000
RelB Abcam ab33907 WB 1:1000
F4/80 Bioss Antibodies bsm-34028M IHC 1:100 IF 1:100
CD86 Bioss Antibodies bs-1035R IF 1:100
Beta Tubulin (HRP conjugated) Bioss Antibodies bsm-52847R WB 1:5000

Flow Cytometry Antibody
CD11c-PC7 eBioScience 25-0114-81 1:50
CD86-PB450 eBioScience 48-0862-80 1:50

Secondary antibody
Anti-rabbit IgG, HRP-linked Antibody Cell Signaling Technology 7074S WB 1:5000
Anti-mouse IgG, HRP-linked Antibody Cell Signaling Technology 7076S WB 1:5000
Goat anti-Rabbit IgG (H+L) Cross-Adsorbed Secondary Antibody, Alexa Fluor™ 488 Thermo Fisher Scientific A-11008 IF 1:2000
Goat anti-mouse IgG (H+L) Secondary Antibody, DyLight™ 488 Thermo Fisher Scientific A-10680 IF 1:2000
Goat anti-Rabbit IgG (H+L) Highly Cross-Adsorbed Secondary Antibody, Alexa Fluor™ 555 Thermo Fisher Scientific A-21429 IF 1:2000
Goat anti-Rabbit IgG (H+L) Cross-Adsorbed Secondary Antibody, Alexa Fluor™ 594 Thermo Fisher Scientific R-37117 IF 1:2000
Goat anti-Rabbit IgG (H+L) Highly Cross-Adsorbed Secondary Antibody, Alexa Fluor™ 647 Thermo Fisher Scientific A-21245 IF 1:2000

Validation

All antibodies used in this study were validated by the supplier as follows:
Rabbit anti-UBE2C IHC bs-8357R Bioss Antibodies
https://www.biossantibodies.com/datasheets/bs-8357R
Rabbit anti-Fibulin1(FBLN1) IHC/IF bs-0809R Bioss Antibodies
https://www.biossantibodies.com/datasheets/bs-0809R
Rabbit anti-CHI3L2 IHC bs-12358R Bioss Antibodies
https://www.biossantibodies.com/datasheets/bs-12358R
Rabbit anti-DKK1 IHC bs-2162R Bioss Antibodies
https://www.biossantibodies.com/datasheets/bs-2162R
Rabbit anti-MSMO1 IHC ab203587 Abcam
https://www.abcam.com/products/primary-antibodies/c-4-methylsterol-oxidasesc4mol-antibody-ab203587.html
Rabbit Anti-Ceruloplasmin IHC bs-2373R Bioss Antibodies
http://www.bioss.com.cn/prolook_03.asp?id=AF08169606003634&pro37=1
Mouse anti-SRGN IHC/IF sc-374657 Santa Cruz
https://www.scbt.com/p/serglycin-antibody-c-11?requestFrom=search
Rabbit anti-Serglycin WB ab156991 (not available) Abcam
https://www.abcam.com/products/primary-antibodies/serglycin-antibody-ab156991.html
Rabbit anti-SRGN WB SAB2103016 Sigma-Aldrich
https://www.sigmaaldrich.cn/CN/zh/product/sigma/sab2103016
Mouse anti-Collagen 1 IHC/IF bs-10423R Bioss Antibodies
https://www.biossusa.com/products/bs-10423r
Rabbit anti-COL2A1 WB #43306 Cell Signaling Technology
https://www.cellsignal.com/products/primary-antibodies/col2a1-e8s2s-rabbit-mab/43306?_=1695578024504&Ntt=col2&tahead=true
Rabbit anti-aggrecan WB #28971 Cell Signaling Technology
https://www.cellsignal.com/products/primary-antibodies/aggrecan-e8b8s-rabbit-mab/28971?_=1695578037273&Ntt=ACAN&tahead=true
Rabbit anti-IL-1 beta WB/IF ab254360 Abcam
https://www.abcam.com/products/primary-antibodies/il-1-beta-antibody-epr23851-127-ab254360.html
Rabbit anti-IL-1 beta IHC ab283818 Abcam
https://www.abcam.com/products/primary-antibodies/il-1-beta-antibody-rm1009-ab283818.html
Rabbit anti-CCL3 WB/IHC/IF ab259372 Abcam
https://www.abcam.com/products/primary-antibodies/macrophage-inflammatory-protein-1-alpha--ccl3--ccl3l1-antibody-epr23751-54-ab259372.html
Rabbit anti-TNF alpha WB ab183218 Abcam
https://www.abcam.com/products/primary-antibodies/tnf-alpha-antibody-epr19147-ab183218.html
Mouse anti-TNF alpha IHC/IF ab1793 Abcam
https://www.abcam.com/products/primary-antibodies/tnf-alpha-antibody-52b83-ab1793.html
Rabbit anti-IKB alpha WB ab32518 Abcam
https://www.abcam.com/products/primary-antibodies/ikb-alpha-antibody-e130-ab32518.html
Rabbit anti-IKB alpha (phospho S36) WB ab133462 Abcam
https://www.abcam.com/products/primary-antibodies/ikb-alpha-phospho-s36-antibody-epr62352-ab133462.html
Rabbit anti-pan-AKT WB ab8805 Abcam
https://www.abcam.com/products/primary-antibodies/pan-akt-antibody-ab8805.html
Rabbit anti-pan-AKT(phospho T308) WB ab8933 Abcam
https://www.abcam.com/products/primary-antibodies/pan-akt-phospho-t308-antibody-ab8933.html
Rabbit anti-Smad2 WB ab40855 Abcam
https://www.abcam.com/products/primary-antibodies/smad2-antibody-ep784y-ab40855.html
Rabbit anti-Smad2(phospho S467) WB ab280888 Abcam
https://www.abcam.com/products/primary-antibodies/smad2-phospho-s467-antibody-epr23681-40-ab280888.html
Rabbit anti-Smad3 WB ab40854 Abcam
https://www.abcam.com/products/primary-antibodies/smad3-antibody-ep568y-ab40854.html
Rabbit anti-Smad3(phospho S423+S425) WB ab52903 Abcam

https://www.abcam.com/products/primary-antibodies/smad3-phospho-s423--s425-antibody-ep823y-ab52903.html
Rabbit anti-ERK1+ERK2 WB ab184699 Abcam
https://www.abcam.com/products/primary-antibodies/erk1--erk2-antibody-epr17526-ab184699.html
Rabbit anti-ERK1(phospho T202)+ERK2(phospho T185) WB ab201015 Abcam
https://www.abcam.com/products/primary-antibodies/erk1-phospho-t202--erk2-phospho-t185-antibody-epr19401-ab201015.html
Rabbit anti-NF-κB p65 WB ab16502 Abcam
https://www.abcam.com/products/primary-antibodies/nf-kb-p65-antibody-ab16502.html
Mouse anti-p-NF-κB p65 WB/IF sc-136548 Santa Cruz
https://www.scbt.com/p/p-nfkappab-p65-antibody-27-ser-536?productCanUrl=p-nfkappab-p65-antibody-27-ser-536&_requestid=2944471
Mouse anti-NFkB p105/p50 WB ab305263 Abcam
https://www.abcam.com/products/primary-antibodies/nfkb-p105--p50-antibody-1298ct792105117133-ab305263.html
Mouse anti-NFκB p52/p100/NFKB2 WB sc-7386 Santa Cruz
https://www.scbt.com/p/nfkappab-p52-antibody-c-5?requestFrom=search
Rabbit anti-c-Rel WB ab133251 Abcam
https://www.abcam.com/products/primary-antibodies/c-rel-antibody-epr25592-ab133251.html
Rabbit anti-Rel B WB ab33907 Abcam
https://www.abcam.com/products/primary-antibodies/rel-b-antibody-ep614y-ab33907.html
Mouse anti-ADGRE1(F4/80) IHC/IF bsm-34028M Bioss Antibodies
http://www.bioss.com.cn/SpeNew01.asp?id=214469&pro37=1&pro33=101&guige01=100ul
Rabbit anti-CD86 IF bs-1035R Bioss Antibodies
https://www.biossantibodies.com/datasheets/bs-1035R
Rabbit anti-beta Tubulin (HRP conjugated) WB bsm-52847R Bioss Antibodies
https://www.biossantibodies.com/datasheets/bsm-52847R
Mouse anti-CD11c PE-Cyanine7 Flow Cytometry 25-0114-81 eBioScience
https://www.thermofisher.cn/cn/zh/antibody/product/CD11c-Antibody-clone-N418-Monoclonal/25-0114-81
Mouse anti-CD86-PB450 Flow Cytometry 48-0862-80 eBioScience
https://www.thermofisher.cn/cn/zh/antibody/product/CD86-B7-2-Antibody-clone-GL1-Monoclonal/48-0862-80
Anti-rabbit IgG, HRP-linked Antibody WB #7074 Cell Signaling Technology
https://www.cellsignal.com/products/secondary-antibodies/anti-rabbit-igg-hrp-linked-antibody/7074?site-search-type=Products&N=4294956287&Ntt=7074s&fromPage=plp&_requestid=3557248
Anti-mouse IgG, HRP-linked Antibody WB #7076 Cell Signaling Technology
https://www.cellsignal.com/products/secondary-antibodies/anti-mouse-igg-hrp-linked-antibody/7076?site-search-type=Products&N=4294956287&Ntt=7076s&fromPage=plp&_requestid=3557493
Goat anti-Rabbit IgG (H+L) Cross-Adsorbed Secondary Antibody, Alexa Fluor™ 488 IF A-11008 Thermo Fisher Scientific
https://www.thermofisher.cn/cn/zh/antibody/product/Goat-anti-Rabbit-IgG-H-L-Cross-Adsorbed-Secondary-Antibody-Polyclonal/A-11008
Goat anti-Mouse IgG, IgM (H+L) Secondary Antibody, Alexa Fluor™ 488 IF A-10680 Thermo Fisher Scientific
https://www.thermofisher.cn/cn/zh/antibody/product/Goat-anti-Mouse-IgG-IgM-H-L-Secondary-Antibody-Polyclonal/A-10680
Goat anti-Rabbit IgG (H+L) Highly Cross-Adsorbed Secondary Antibody, Alexa Fluor™ 555 IF A-21429 Thermo Fisher Scientific
https://www.thermofisher.cn/cn/zh/antibody/product/Goat-anti-Rabbit-IgG-H-L-Highly-Cross-Adsorbed-Secondary-Antibody-Polyclonal/A-21429
Goat anti-Rabbit IgG (H+L) Cross-Adsorbed Secondary Antibody, Alexa Fluor™ 594 IF R-37117 Thermo Fisher Scientific
https://www.thermofisher.cn/cn/zh/antibody/product/Goat-anti-Rabbit-IgG-H-L-Cross-Adsorbed-Secondary-Antibody-Polyclonal/R37117
Goat anti-Rabbit IgG (H+L) Highly Cross-Adsorbed Secondary Antibody, Alexa Fluor™ 647 IF A-21245 Thermo Fisher Scientific
https://www.thermofisher.cn/cn/zh/antibody/product/Goat-anti-Rabbit-IgG-H-L-Highly-Cross-Adsorbed-Secondary-Antibody-Polyclonal/A-21245

Eukaryotic cell lines

Policy information about cell lines and Sex and Gender in Research

Cell line source(s)	The RAW264.7 cells in this study was obtained from Sunncell Biotech (Cat#. SNL-112, Wuhan, China).
Authentication	The Raw264.7 cells were authenticated by STR authentication(Cell line STR authentication report is provided in Supplementary Table S7) in Sunncell Biotech before obtained, and subsequent authentication was conducted through morphology checks and growth curve analysis during the cultivation process.
Mycoplasma contamination	Cell lines were routinely tested and found negative for mycoplasma infection by the vender.
Commonly misidentified lines (See ICLAC register)	None of the cell lines used in this study is found in the database of commonly misidentified cell lines.

Animals and other research organisms

Policy information about studies involving animals; ARRIVE guidelines recommended for reporting animal research, and Sex and Gender in Research

Laboratory animals	Animal study was conducted by 7-8 weeks Srgn knockout C57BL/6 (n = 5 per group) , which were purchased from Cyagen Biosciences Inc (Suzhou, China). All mice were raised in SPF environment under constant temperature (23–25°C) and humidity (50%) with a 12-hour light/12-hour dark circadian cycle.<br>the method of Srgn knockout mice construction: the gRNA for the mouse SRGN gene and Cas9 mRNA were coinjected into fertilized mouse eggs to generate targeted knockout offspring. F2 founder animals were identified by PCR (Supplementary Fig. S5 a-f), followed by sequencing analysis. Heterozygous mice were then bred to assess germline transmission and F3 animal generation.
Wild animals	This study did not involved wild animals
Reporting on sex	No sex- and gender-based analyses have been performed.
Field-collected samples	The study did not involve samples collected from the field.
Ethics oversight	Protocol of animal model of intradiscal injection was approved by The Institutional Animal Care and Use Committee (IACUC) at The First Affiliated Hospital of Sun Yat-sen University (No. [2020]017).

Note that full information on the approval of the study protocol must also be provided in the manuscript.

Flow Cytometry

Plots

Confirm that:

- [x] The axis labels state the marker and fluorochrome used (e.g. CD4-FITC).
- [x] The axis scales are clearly visible. Include numbers along axes only for bottom left plot of group (a 'group' is an analysis of identical markers).
- [x] All plots are contour plots with outliers or pseudocolor plots.
- [x] A numerical value for number of cells or percentage (with statistics) is provided.

Methodology

Sample preparation	The treated RAW264.7 cells were concentrated, washed with PBS twice. After washing, cells were incubated with fluorescence-labeled surface Abs against CD11c and CD86 (eBioscience) for 30 min at 4°C.Fluorescence signals were detected using the Beckman CytoFLEX Flow Cytometer.
Instrument	Cytoflex, Beckman Coulter, Inc.
Software	CytExpert analyzer
Cell population abundance	CytExpert analyzer for data collection. FlowJo software for data analysis.
Gating strategy	All samples were initially gated using forward scatter and side scatter to identify events corresponding to cells or, alternatively by CD11c and CD86 to identify events corresponding to M1 macrophages. The follow gating steps are presented in principal and supplementary figures.

- [x] Tick this box to confirm that a figure exemplifying the gating strategy is provided in the Supplementary Information.
